# IVIM-based habitat imaging for non-invasive assessment of Ki-67 proliferation status in cervical cancer

**DOI:** 10.3389/fonc.2026.1885498

**Published:** 2026-10-14

**Authors:** Wenjing Yue, Hui Tian, Xiaofei Cai, Bofeng Yu, Yunqing Ban, Qiqi Kong

**Affiliations:** 1 Department of Radiology, The Fifth Affiliated Hospital of Xinjiang Medical University, Urumqi, Xinjiang, China; 2 Department of Cardiovascular Surgery, People’s Hospital of Xinjiang Uygur Autonomous Region, Urumqi, Xinjiang, China

**Keywords:** cervical cancer, diffusion-weighted imaging - DWI, habitat imaging, imaging biomarker, intravoxel incoherent motion (IVIM), Ki-67, magnetic resonance imaging, tumor heterogeneity (TH)

## Abstract

**Objective:**

This study aimed to evaluate whether voxel-level IVIM-based habitat analysis supports exploratory pretreatment stratification of Ki-67 proliferation status in cervical cancer.

**Materials and methods:**

Between January 2019 and June 2025, medical records of 149 patients with histopathologically confirmed cervical cancer were initially screened. Before image-quality assessment, 49 were excluded because IVIM MRI had been performed after chemoradiotherapy (n = 5), cervical biopsy sampling (n = 21), or lesion excision (n = 10), or because no pathological Ki-67 immunohistochemistry result was available (n = 13). Of the remaining 100 patients, 17 were excluded because of severe image artefacts (n = 11) or lesions too small for reliable ROI delineation (n = 6), leaving 83 patients for analysis. Patients were stratified into high Ki-67 (Ki-67 >70%, n = 38) and low/equal Ki-67 (Ki-67 ≤70%, n = 45) groups. Solid-tumour regions were analysed with patient-level K-means clustering using normalised D, f, and ADC features; D* was excluded from clustering because of voxel-level fitting instability. Habitat labels were harmonised according to quantitative parameter profiles and treated as image-defined phenotypes. Habitat-derived features, conventional ROI parameters, and logistic models were evaluated for Ki-67 stratification.

**Results:**

Hab1 ADC was lower in the high- than in the low/equal-Ki-67 group (median [Q1–Q3], 0.947 [0.862–1.161] vs. 1.257 [1.113–1.455] × 10^−3^ mm^2^/s; *P* < 0.001; BH *q* = 2.04×10^−6^), whereas Hab3 volume proportion was higher (0.396 [0.341–0.488] vs. 0.279 [0.190–0.390]; *P* = 0.002; BH *q* = 0.006). Hab1 ADC outperformed conventional ROI ADC for identifying high Ki-67 status [AUC, 0.826 (95% CI, 0.726–0.909) vs. 0.563 (95% CI, 0.429–0.695); paired DeLong *P* < 0.001]. The combined Hab1 ADC and Hab3 volume proportion model showed the numerically highest discrimination (apparent AUC, 0.877; 95% CI, 0.794–0.938), but this numerical improvement over Hab1 ADC alone was not statistically significant (paired DeLong *P* = 0.056). Fixed-feature leave-one-out validation of the combined model yielded an AUC of 0.855 (95% CI, 0.771–0.923) and a Brier score of 0.157 (95% CI, 0.112–0.204).

**Conclusion:**

IVIM-based habitat analysis showed exploratory potential for non-invasive Ki-67 stratification by isolating a low-diffusivity imaging compartment otherwise obscured by conventional whole-tumour averaging. The combined habitat model should be interpreted as a research-stage imaging biomarker, not a clinical decision tool, and requires prospective external validation before clinical translation.

## Introduction

Cervical cancer remains a major cause of cancer morbidity and mortality worldwide, with a disproportionate burden in low- and middle-income settings (1). Clinical management is guided principally by stage and established clinicopathological factors (2). Ki-67 is an immunohistochemical marker of proliferative activity that has prognostic relevance in cervical cancer (3). However, a tissue measurement obtained from a biopsy or surgical specimen may not fully represent a spatially heterogeneous tumour. Cervical cancers may contain coexisting cellular and microenvironmental states, including hypoxia-related variation, that can contribute to treatment resistance and disease evolution (4, 5). A whole-tumour imaging approach may therefore provide information complementary to pathological sampling.

Intravoxel incoherent motion (IVIM) MRI uses a biexponential signal model to estimate diffusion-related (D) and perfusion-related (f and D*) components (6). IVIM parameters have been investigated for functional characterization of cervical cancer, although their values depend on acquisition and fitting choices (7, 8). A previous IVIM-DWI study found lower D in tumours with higher Ki-67 expression and improved discrimination when diffusion and texture information were combined (9). Other recent cervical studies have linked non-Gaussian diffusion or combined DWI/DCE parameters with Ki-67 (10, 11). These findings support diffusion MRI as a candidate marker of proliferative heterogeneity, while also indicating that global summary values may obscure spatially localised signal patterns.

Habitat imaging addresses this limitation by partitioning multiparametric images into voxel groups with similar signal profiles; these groups remain image-defined unless validated by spatially matched pathology (12). In cervical cancer, habitat radiomics has been studied for lymphovascular-space invasion and treatment response (13, 14). Recent prospective and dual-centre MRI studies have extended this framework to additional preoperative risk assessments (15, 16). Nevertheless, whether IVIM-derived habitats can improve non-invasive assessment of Ki-67 remains insufficiently studied.

The present study therefore applied K-means clustering to cervical-cancer IVIM images to construct image-defined habitat maps. We aimed to (i) evaluate whether Hab1 ADC extracted from the low-diffusivity habitat outperforms conventional whole-tumour ROI ADC for predicting Ki-67 proliferation status; (ii) characterise whether the Hab3 volume proportion adds predictive information; (iii) assess the performance and fixed-feature internal validation of a combined habitat model; and (iv) explore the feasibility of deriving unsupervised imaging phenotypes from habitat features. These analyses were designed for exploratory pretreatment risk stratification and require external validation before clinical translation.

## Materials and methods

### Study design and participants

This retrospective single-centre observational study was conducted at the Fifth Affiliated Hospital of Xinjiang Medical University. The study was approved by the institutional Ethics Committee (Approval No. XYDWFYLSk-2025-14), which waived the requirement for individual written informed consent because the study used retrospective, anonymised data. The study population comprised patients with histopathologically confirmed primary cervical cancer identified between January 2019 and June 2025. Eligibility required baseline 3.0-T pelvic MRI, including multi-b-value IVIM-DWI, acquired within 2 weeks before treatment and before cervical biopsy sampling, lesion excision, or chemoradiotherapy; an available pathological Ki-67 immunohistochemistry result; and complete records for FIGO stage and histological subtype. Of 149 initially screened patients, 49 were excluded before image-quality assessment because IVIM MRI had been performed after chemoradiotherapy (n = 5), after cervical biopsy sampling (n = 21), or after lesion excision (n = 10), or because no pathological Ki-67 immunohistochemistry result was available (n = 13). The remaining 100 patients underwent detailed image-quality and ROI-eligibility assessment. Seventeen were excluded because of severe motion, susceptibility, metallic implant, or other image artefacts that prevented reliable measurement (n = 11), or because the maximum tumour diameter was <5 mm (n = 6), leaving 83 patients for analysis. The participant-selection pathway is summarised in **Figure 1**, and the cohort characteristics are presented in **Table 1**.

**Figure 1:**
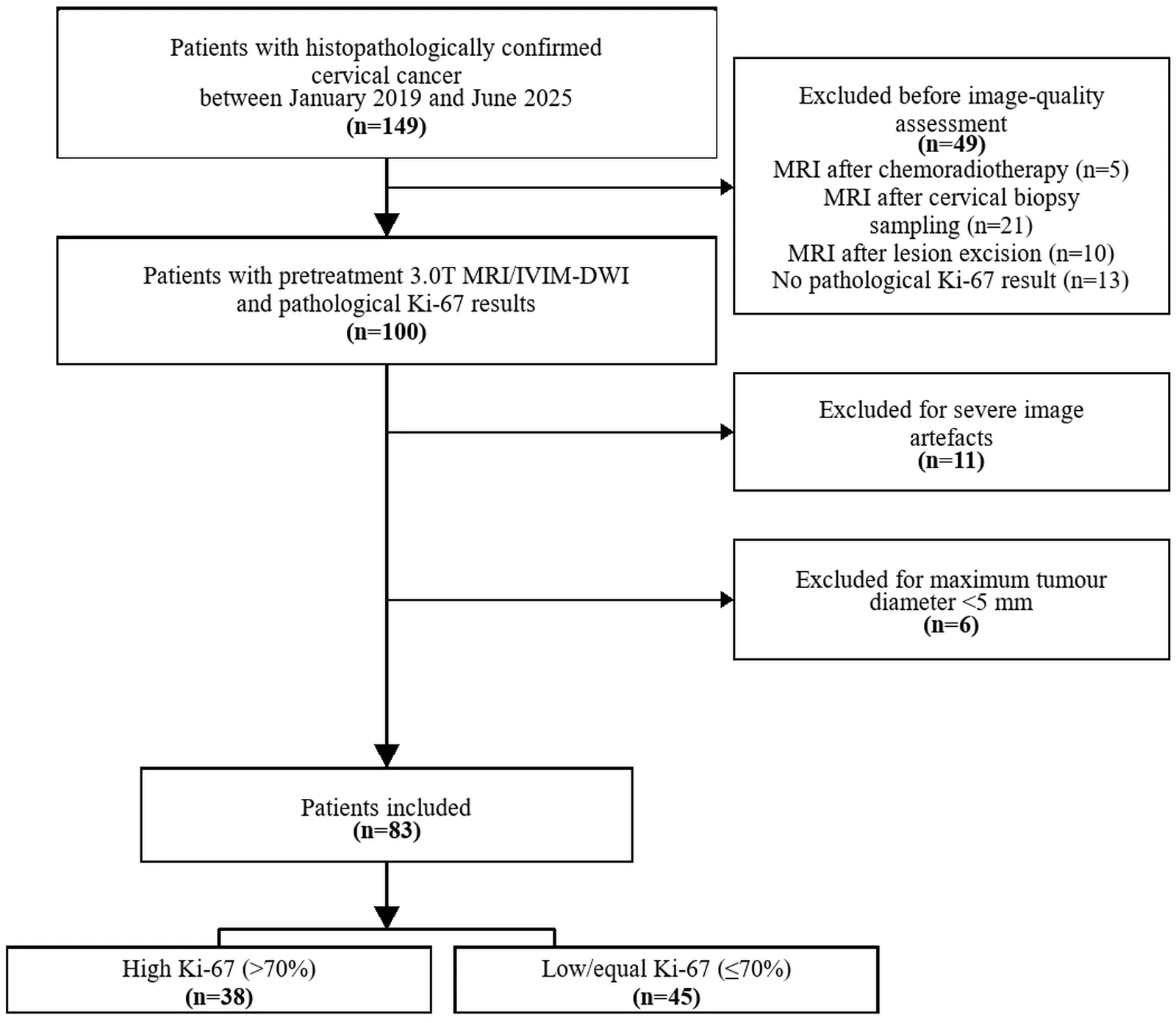
Participant selection flow diagram. Hospital records identified 149 patients with histopathologically confirmed primary cervical cancer. Before image-quality assessment, 49 were excluded because IVIM MRI was performed after chemoradiotherapy (n = 5), cervical biopsy sampling (n = 21), or lesion excision (n = 10), or because no pathological Ki-67 immunohistochemistry result was available (n = 13). Of the remaining 100 patients, 11 were excluded because of severe image artefacts and 6 because the maximum tumour diameter was <5 mm, leaving 83 patients for analysis (high Ki-67, n = 38; low/equal Ki-67, n = 45).

**Table 1:** Clinicopathological characteristics of the study cohort.

Characteristic	High Ki-67 (>70%) n = 38	Low/equal Ki-67 (≤70%) n = 45	*P* value
Age (years), mean ± SD	57.0 ± 13.0	53.0 ± 12.3	0.162
FIGO stage			0.392
I	20 (52.6)	22 (48.9)	
II	8 (21.1)	9 (20.0)	
III	8 (21.1)	14 (31.1)	
IV	2 (5.3)	0 (0.0)	
Histological subtype			0.646
Squamous-cell carcinoma	26 (68.4)	28 (62.2)	
Adenocarcinoma	12 (31.6)	17 (37.8)	
Histological differentiation			< 0.001
Well differentiated	4 (10.5)	20 (44.4)	
Moderately differentiated	12 (31.6)	22 (48.9)	
Poorly differentiated	22 (57.9)	3 (6.7)	
Maximum tumour diameter (cm), median [Q1–Q3]	5.54 [4.11–7.28]	6.17 [4.48–7.61]	0.519
Tumour volume (cm^3^), median [Q1–Q3]	15.151 [7.899–44.008]	20.895 [10.225–45.195]	0.684

Values are n (%), mean ± SD, or median [Q1–Q3]. Age was compared using a two-sample t-test; FIGO stage, histological subtype, and differentiation were compared using exact contingency-table tests; and tumour diameter and volume were compared using two-sided Mann–Whitney U tests. FIGO, International Federation of Gynecology and Obstetrics.

No *a priori* sample-size calculation was performed. The study size comprised all retrievable cervical cancer cases from the gynaecology outpatient service that were screened in chronological order that met the eligibility and image-quality criteria during the study period. Because eligible patients with baseline 3.0-T IVIM-DWI were uncommon, all eligible cases with complete analysable imaging and pathological Ki-67 data were retained.

### MRI acquisition and preprocessing

MRI was performed on a 3.0-T GE Discovery MR750 system (GE Healthcare, Milwaukee, WI, USA) with an eight-channel phased-array torso coil. Axial IVIM-DWI was acquired using a fat-suppressed single-shot spin-echo echo-planar imaging sequence with b values of 0, 50, 100, 200, 400, 600, 800, and 1,000 s/mm^2^. The corresponding numbers of excitations were 1, 1, 1, 1, 1, 2, 4, and 4, respectively. Other acquisition parameters were TR = 4,500 ms, TE = 58.3–60.4 ms, flip angle = 90°, acquisition matrix=128×128, reconstructed matrix = 256×256, reconstructed in-plane resolution = 1.25–1.56 mm, phase field of view = 80%, slice thickness = 4.5–5.5 mm, spacing between slice centres = 5.5–7.0 mm, receiver bandwidth = 1,953.12 Hz/pixel, and in-plane parallel-imaging factor = 2 (ASSET). Acquisition time was approximately 198 s. Fat saturation was confirmed from the DICOM scan option FS. The scanner exported one scalar magnitude volume for each b value; direction-resolved diffusion images or diffusion-tensor metrics were not used. Complete acquisition, reconstruction, and preprocessing details are provided in **Supplementary Table S1**.

DICOM images were converted to NIfTI format with dcm2niix (v1.0.20250505), and b values were verified against the exported sidecars. The eight b-value volumes were acquired as a single four-dimensional IVIM series, and the parameter maps were generated directly in that native grid. Therefore, no additional inter-b-value registration, interpolation, spatial smoothing, or resampling was applied before fitting. No additional susceptibility-distortion correction was performed. The tumour ROI and all derived IVIM maps were analysed in the same native EPI voxel grid, so no cross-sequence registration was required for habitat construction.

Voxel-wise fitting was implemented in a custom Python/PyTorch script. Voxels with a b = 0 signal intensity of >60 scanner units were processed, and the measured b = 0 signal was fixed as S0. The D, f, and D* parameters were estimated simultaneously from all eight b values by minimizing the unweighted mean squared error in the signal domain using the Adam optimiser (1,000 iterations; learning rate, 0.02). The biexponential model was;


S(b)/S(0)=(1−f)exp(−bD)+fexp(−bD*),

where *D* and D* are expressed in mm^2^/s and *f* is a fraction. Optimisation was initialised in the transformed parameter space at raw values of 0, −3, and −7 for *f*, D*, and *D*, respectively. The transformations *f* = sigmoid(*raw_f*), D* = exp(raw_D*), and *D* = exp(*raw_D*) corresponded to starting values of *f* = 0.50, D* = 0.0498 mm^2^/s, and *D* = 0.000912 mm^2^/s and enforced 0 < *f* < 1, D* > 0, and *D* > 0. No hard upper bounds or D*>*D* ordering constraint were imposed during optimisation. Conventional ADC was estimated separately by log-linear regression across all eight *b* values; an auxiliary high-*b*-value monoexponential diffusion map was generated using *b* ≥ 200 s/mm^2^. A numerical constant of 10^−6^ was added before logarithmic transformation to avoid log(0). No explicit Rician or non-central-χ noise-floor correction was applied to the simultaneous biexponential fit.

The optimiser used a fixed iteration count rather than a separate convergence status flag. Operational voxel-level quality control was therefore based on the input-signal mask and post-fit validity checks. Before clustering, only finite estimates satisfying 0 < D < 10 and 0 < ADC <10 (both in 10^−3^ mm^2^/s) and 0≤f ≤ 1 were retained; patients were required to have at least 50 valid tumour voxels. D* was retained as an auxiliary fitted map but was not used as a clustering feature because of its recognised fitting instability (8, 17).

### Tumour ROI delineation and inter-observer reproducibility

The solid-tumour ROI was manually delineated on high-b-value DWI images by a radiologist with 10 years of pelvic MRI experience. Gross necrotic regions were excluded, and a one- to two-voxel margin was used to reduce partial-volume effects. Each ROI contained more than 50 analysable voxels. A second radiologist with 15 years of pelvic MRI experience independently repeated the delineation and measurement in all 83 study patients. Agreement was quantified using two-way random-effects, absolute-agreement, single-measure intraclass correlation coefficients (ICCs) with bootstrap 95% confidence intervals. Complete variable-level ICC estimates are provided in **Supplementary Table S2**.

### Patient-level habitat construction and label harmonisation

For each patient, valid voxels within the solid-tumour ROI were represented by their D, f, and ADC values. These three features were standardised separately within each patient and clustered using K-means with k = 3, k-means++ initialisation, random_state = 42, and n_init = 10 (18, 19). Spatial coordinates were not included in the clustering; therefore, the resulting classes represent groups of voxels with similar IVIM profiles and were not assumed to be spatially contiguous biological regions.

Because patient-level standardisation makes raw cluster indices arbitrary, labels were harmonised using a locked, outcome-blind profile score evaluated across all six possible assignments of the three raw clusters. For a candidate assignment of the three raw clusters to Hab1, Hab2, and Hab3, respectively, the score was;


−5|D(Hab1)−1.0|+100f(Hab2)+10D(Hab3)−P,

where *P* was 200 if D(Hab1)<0.55, 50 if D(Hab1)>1.8, 500 if f(Hab3)>f(Hab2), and 50 if ADC(Hab3)<ADC(Hab1); otherwise the corresponding penalty was zero. D and ADC were expressed in 10^−3^ mm^2^/s and f as a fraction. The highest-scoring assignment was selected automatically for each patient; Ki-67 and all clinicopathological variables were unavailable at this step. At the cohort level, the assigned classes were described as low-diffusivity (Hab1), high-perfusion-fraction (Hab2), and high-diffusivity (Hab3) imaging habitats. These are cohort-level imaging-profile descriptors; they do not require every parameter to be ordered identically in every patient and are not direct histological diagnoses. The cohort-level parameter profiles used for this harmonisation are summarised in **Table 2**.

**Table 2:** Cohort-level parameter profiles used for image-defined habitat label harmonisation.

Image-defined habitat	ADC (×10^−3^ mm^2^/s), mean ± SD	D (×10^−3^ mm^2^/s), mean ± SD	f, mean ± SD	Quantitative label rationale
Hab1 (low diffusivity)	1.173 ± 0.267	0.968 ± 0.279	0.240 ± 0.050	Lowest ADC with relatively low D
Hab2 (high perfusion fraction)	1.420 ± 0.370	0.870 ± 0.360	0.530 ± 0.114	Highest f
Hab3 (high diffusivity)	1.592 ± 0.688	1.418 ± 0.713	0.240 ± 0.060	Highest ADC and D

Labels describe quantitative image-parameter profiles used to harmonise K-means clusters across patients. They do not infer direct histological identity. ADC, apparent diffusion coefficient; D, true diffusion coefficient; f, perfusion fraction; Hab, image-defined habitat.

For each patient, mean D, f, and ADC and the voxel-volume proportion were calculated for each harmonised class. The primary imaging predictors were Hab1 ADC and the Hab3 volume proportion. Conventional whole-tumour ADC was retained as the ROI-averaged comparator. The Hab2 volume proportion was evaluated only in an exploratory sensitivity model.

### Robustness analyses of habitat construction

Segmentation robustness was evaluated at two perturbation severities. As a deterministic stress test, a one-pixel in-plane erosion and dilation were applied independently to every axial ROI slice with a four-connected cross-shaped structuring element; through-plane morphology was avoided because of the anisotropic voxel dimensions. Because a full one-pixel operation represented a large proportional volume change in some lesions, a complementary mild analysis contracted or expanded each ROI by exact target-volume changes of −5%, +5%, −10%, and +10%. For each patient and condition, 30 fixed Monte Carlo realisations selected patches from the current four-connected in-plane boundary using a Gaussian-smoothed random field (σ = 1.25 pixels), while preserving every originally occupied axial slice. The complete locked habitat pipeline was rerun for every perturbed ROI with the original validity filter, patient-level standardisation, k = 3, random seed, initialisation count, and label-harmonisation rule. The patient median across Monte Carlo realisations was used for inference; realisations were not treated as independent observations.

Initialisation stability was evaluated by repeating K-means with 20 alternative random states (0–19), retaining n_init = 10 for every fit and using random state 42 as the locked reference. Partition agreement was quantified with the adjusted Rand index (ARI), semantic voxel agreement, and habitat-specific Dice coefficients. Changes in Hab1 ADC and the Hab3 volume proportion, and the performance of the unchanged two-feature logistic model, were also evaluated; each seed-specific model was assessed with fixed-feature leave-one-out cross-validation.

Sensitivity to the clustering representation was assessed with four outcome-blind feature sets (ADC+f, D+f, D+ADC+f, and the first principal component of D and ADC combined with f) and four scaling methods (patient-level z-score, patient-level median/interquartile-range scaling, cohort-level z-score, and no scaling). All remaining settings were fixed. Cluster-number sensitivity was evaluated at k = 2, 3, 4, and 5 using the same patient-level D+ADC+f z-score representation. Silhouette coefficients were calculated within each patient, using the same fixed subsample of at most 2,000 voxels across k values. For direct downstream comparisons, k = 4 and k = 5 clusters were merged to the three locked semantic classes by outcome-blind maximum voxel overlap; k = 2 was reported only as an exploratory two-class surrogate because it cannot instantiate three nonempty habitats. Across robustness analyses, paired patient-level bootstrap intervals, Wilcoxon signed-rank tests, and paired DeLong tests were used as appropriate, with Holm correction within each defined comparison family. Detailed settings and patient-level results are provided in **Supplementary Tables S4–S7** and **Supplementary Figures S1–S4**.

### Pathological assessment and outcome definition

Primary cervical cancer was confirmed by cervical biopsy or surgical pathology, and Ki-67 was evaluated on the available diagnostic biopsy or resection material. Immunohistochemical staining used the streptavidin–peroxidase method. Ki-67 was detected using a mouse anti-human Ki-67 monoclonal antibody (clone MIB-1; OriGene Wuxi Biotechnology Co., Ltd., Wuxi, China; distributed by ZSGB-BIO, Beijing, China; working dilution, 1:100) on a BenchMark ULTRA automated immunohistochemistry platform (Ventana Medical Systems/Roche Diagnostics, Tucson, AZ, USA). The pathology and imaging assessments were conducted independently, with the assessors mutually blinded. A senior pathologist, blinded to the IVIM measurements, habitat maps, and model outputs, selected the highest-density hotspot at low magnification and counted immunoreactive tumour-cell nuclei among 500 consecutive tumour cells at ×400 magnification; the final Ki-67 index was the percentage of nuclei staining positive. The radiologists were blinded to the Ki-67 result during ROI delineation and habitat feature extraction. The archived methods and analytical file do not document a second independent pathology rescore; pathology-reader reproducibility was therefore not estimated in this retrospective reanalysis.

Specimen source and MRI-to-pathology timing were verified case by case from the completed pathology-timing audit table. Ki-67 assessment used diagnostic biopsy material in 49 patients and surgical resection material in 34 patients. Across all 83 patients, the MRI-to-pathology interval was a median of 1 day [Q1–Q3, 0–3 days] with a range of 0–7 days. The interval was a median of 1 day [Q1–Q3, 0–2 days] for diagnostic biopsy specimens and a median of 2.5 days [Q1–Q3, 0.25–5 days] for surgical resection specimens; the corresponding ranges were 0–7 days in both groups. The recorded interval matched the recalculated date difference in all 83 rows, and no negative intervals were identified. An exploratory sensitivity model was added with specimen type (surgical resection versus diagnostic biopsy) as a fixed covariate. By the eligibility criteria, every included MRI examination was also performed within 2 weeks before treatment. Ki-67 was analysed both as a continuous percentage and as the retained exploratory binary outcome: high Ki-67 (>70%) versus low/equal Ki-67 (≤70%). The >70% threshold had been selected from the cohort distribution in the submitted analysis; no external clinical or pathological justification was identified. It was retained for comparability with the submitted results and to avoid ambiguity in cases recorded at exactly 70%, but was not treated as a clinically validated treatment threshold. Continuous Ki-67 results were therefore reported prominently, and sensitivity analyses used >60%, >80%, and ≥70% definitions.

### Statistical analysis

All analyses were performed at the patient level, with one row per patient. The working table contained 83 unique patient identifiers and no missing values in the analysed variables; therefore, no imputation was performed. Continuous variables were summarised as mean ± standard deviation or median [Q1–Q3], as appropriate. Shapiro–Wilk tests and interquartile-range checks were used to describe distributional features. Group differences in imaging and volume features were assessed using two-sided Mann–Whitney U tests with rank-biserial effect sizes. The eight-variable imaging-and-volume comparison family (Hab1 ADC, D, and f; Hab3 and Hab2 volume proportions; Hab2 ADC; conventional ROI ADC; and tumour volume) was adjusted using the Benjamini–Hochberg false-discovery-rate procedure; both raw *P* and *q* values are reported in **Table 3**. Categorical variables were compared using exact tests where sparse cells were present. FIGO stage was consolidated into stages I–IV; cases with nodal disease coded N1 were assigned to stage III to reproduce the submitted stage table.

**Table 3:** Between-group comparison of imaging and volume features with Benjamini–Hochberg correction.

Parameter	High Ki-67 (n = 38), median [Q1–Q3]	Low/equal Ki-67 (n = 45), median [Q1–Q3]	Mann– Whitney U	Rank-biserial effect	Raw *P*	BH *q*
Hab1 ADC (×10^−3^ mm^2^/s)	0.947 [0.862–1.161]	1.257 [1.113–1.455]	298	−0.651	3.65×10^−7^	2.04×10^−6^
Hab1 D (×10^−3^ mm^2^/s)	0.731 [0.650–0.985]	1.044 [0.903–1.269]	305	−0.643	5.10×10^−7^	2.04×10^−6^
Hab1 f	0.231 [0.217–0.239]	0.235 [0.226–0.257]	600	−0.298	0.020	0.032
Hab3 volume proportion	0.396 [0.341–0.488]	0.279 [0.190–0.390]	1188	0.389	0.002	0.006
Hab2 volume proportion	0.120 [0.100–0.151]	0.149 [0.124–0.192]	529	−0.381	0.003	0.006
Hab2 ADC (×10^−3^ mm^2^/s)	1.449 [1.184–1.745]	1.351 [1.061–1.610]	1003	0.173	0.178	0.237
Conventional ROI ADC (×10^−3^ mm^2^/s)	1.236 [1.098–1.503]	1.369 [1.197–1.451]	748	−0.125	0.330	0.378
Tumour volume (cm^3^)	15.151 [7.899–44.008]	20.895 [10.225–45.195]	810	−0.053	0.684	0.684

High Ki-67 was defined as >70%; low/equal Ki-67 as ≤70%. Each row used a two-sided Mann–Whitney U test. BH *q-*values control the false-discovery rate across the eight listed imaging-and-volume comparisons. Positive rank-biserial effects indicate higher ranks in the high-Ki-67 group. ADC, apparent diffusion coefficient; BH, Benjamini–Hochberg; Hab, image-defined habitat; ROI, region of interest.

Spearman rank correlations were used for continuous Ki-67 associations, with bootstrap 95% confidence intervals. A multivariable ordinary least-squares model was fitted with HC3 heteroscedasticity-robust standard errors; predictors included Hab1 ADC, Hab3 volume proportion, poor differentiation, FIGO stage, histological subtype, age, and log-transformed tumour volume. Effects for ADC and habitat proportions were scaled per 0.1 × 10^−3^ mm^2^/s and per 10 percentage points, respectively.

No class weighting, over-sampling, under-sampling, or synthetic-sample method was used. All 83 patients contributed once to each applicable analysis. Firth bias reduction was used for sparse covariate cells; it was not used as a class-balancing procedure.

For binary outcome modelling, conventional ROI ADC, Hab1 ADC alone, and the originally reported imaging model (Hab1 ADC plus Hab3 volume proportion) were fitted using logistic regression. To evaluate pathological confounding and incremental value, we fitted three fixed comparison models using Firth bias-reduced logistic regression because of sparse pathological subgroups (20): (i) an imaging-only model containing Hab1 ADC and Hab3 volume proportion; (ii) a clinicopathological model containing histological differentiation (poorly and well differentiated, with moderately differentiated tumours as the reference), ordinal FIGO stage, histological subtype, age, and log-transformed tumour volume; and (iii) a combined model containing both predictor sets. Hab1 ADC was expressed per 0.1 × 10^−3^ mm^2^/s, Hab3 volume proportion per absolute 0.1 increment, and age per decade. Predictors were fixed before model refitting, and no automated variable selection was performed. Full equations, coefficient codings, probability thresholds, and operating characteristics are provided in **Supplementary Table S8**. Because Hab1 ADC and Hab1 D were strongly correlated, variance inflation factors and a two-variable principal component analysis were used as patient-level redundancy checks; ADC and D were not entered simultaneously into the primary downstream regression model.

For the secondary outcome-blind imaging-phenotype analysis shown in **Figure 2**, Hab1 ADC, Hab1 *D*, and Hab3 volume proportion were standardised across all 83 patients with scikit-learn StandardScaler (feature means of 0 and population variances of 1). The two-cluster solution (*k* = 2), selected in the original exploratory analysis by inspection of the within-cluster sum-of-squares elbow before Ki-67 was examined, was fitted in the full standardised three-feature space using K-means with *k*-means++ initialisation, n_init = 10, max_iter = 300, tol = 1×10^−4^, and random_state = 42. PCA with n_components = 2 was fitted independently to the same standardised matrix only for two-dimensional display; PC1 and PC2 explained 66.3% and 33.1% of total variance, respectively. The resulting K-means labels (Cluster 0, *n* = 34; Cluster 1, *n* = 49) were overlaid on the PCA score plot. Each 95% ellipse was calculated from the within-cluster mean and covariance of the two PC scores and scaled by the 95th percentile of the chi-square distribution with two degrees of freedom (5.991). The final K-means solution had an inertia of 144.934 and converged after 9 iterations. No Ki-67 value or clinicopathological variable entered the standardisation, PCA, cluster fitting, or cluster-number selection. This secondary analysis was descriptive and was not used to define, train, or validate the primary Ki-67 model.

**Figure 2:**
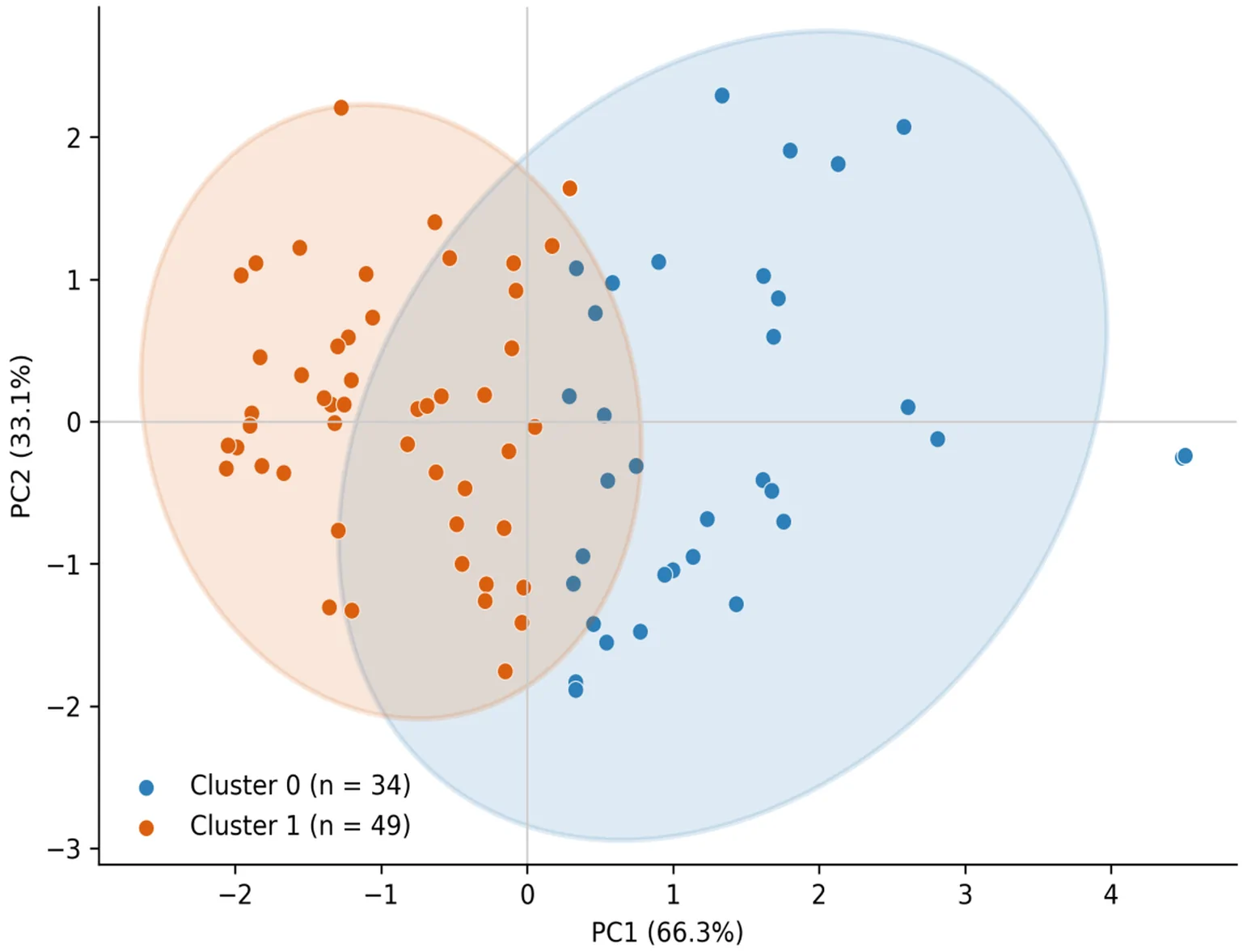
PCA display of the secondary outcome-blind patient-level phenotypic clustering. K-means was fitted to cohort-standardised Hab1 ADC, Hab1 *D*, and Hab3 volume proportion in the full three-feature space (*k* = 2, *k*-means++ initialization, n_init = 10, random_state = 42); PCA was used only for two-dimensional visualization (PC1, 66.3%; PC2, 33.1%). Blue and orange points denote Cluster 0 (*n* = 34) and Cluster 1 (*n* = 49), respectively; ellipses are covariance-based 95% data-distribution regions. This exploratory analysis was not used to train or validate the primary Ki-67 model.

Discrimination was summarised by ROC AUC with 1,000-bootstrap 95% confidence intervals. Paired DeLong tests were used only to compare correlated ROC AUCs (21). Sensitivity, specificity, positive predictive value, negative predictive value, accuracy, F1 score, and Brier score were calculated at the cohort-derived Youden cut-off, with bootstrap 95% confidence intervals, and are reported as apparent, in-sample estimates rather than clinical operating characteristics. Fixed-feature leave-one-out cross-validation (LOOCV) generated out-of-fold probabilities; AUC, calibration intercept, calibration slope, and Brier score were reported with patient-level bootstrap 95% confidence intervals. Paired bootstrap differences quantified the change in AUC and Brier score after adding imaging to the clinicopathological model. Bootstrap optimism correction used 400 resamples for the submitted imaging models and 500 resamples for the three-model Firth comparison. A 1,000-permutation label test refitted the combined imaging model after shuffling the outcome. The voxel-to-habitat feature-generation pathway was locked but was not repeated inside each model-resampling split; the validation is therefore described as fixed-feature internal validation, not fully nested validation. No external validation cohort was available. Formal fairness or subgroup-performance analyses were not prespecified: all participants were women with cervical cancer, and ethnicity, socioeconomic status, and other potentially relevant sociodemographic variables were not retained in the analytical dataset. Two-sided *P* < 0.05 was considered statistically significant unless otherwise specified. The main IVIM fitting environment used Python 3.9 and PyTorch; the exact PyTorch patch version was not retained in the archived environment metadata. The revision analyses were executed in Python 3.13.9 with NumPy 2.3.5, pandas 2.3.3, SciPy 1.15.3, scikit-learn 1.8.0, and statsmodels 0.14.6. Generative-AI assistance used during manuscript preparation is described in the Generative AI Statement; it was limited to language editing, formatting checks, reference-style consistency, and drafting and review of analysis code. The authors executed and independently verified all final analyses, source data, figures, and manuscript text; no generative AI system was used to generate, alter, or fabricate patient data or medical images.

Reporting was organised according to STROBE, TRIPOD+AI, and the applicable items of CLAIM 2024 (22–24). Completed checklists are provided as **Supplementary Files**.

## Results

### Cohort characteristics and reproducibility

The final cohort comprised 83 patients (mean age, 54.8 ± 12.7 years), including 54 squamous cell carcinomas and 29 adenocarcinomas. Histological differentiation was classified as well differentiated in 24 patients, moderately differentiated in 34, and poorly differentiated in 25. The FIGO stage distribution was stage I (n = 42), stage II (n = 17), stage III (n = 22), and stage IV (n = 2). In all, 38 patients had high Ki-67 (>70%), and 45 had Ki-67 ≤70%. Age, consolidated FIGO stage, and histological subtype did not differ significantly between Ki-67 groups, whereas poor differentiation was markedly more frequent in the high-Ki-67 group (22/38 [57.9%] versus 3/45 [6.7%]; *P* < 0.001).

Inter-observer agreement was good to excellent across the measured variables, with ICC values ranging from 0.779 to 0.999. ICCs were 0.979 for Hab1 ADC, 0.983 for the Hab3 volume proportion, and 0.980 for conventional whole-tumour ADC. The lowest ICC was observed for the f value of the high-diffusivity habitat (0.779; 95% bootstrap CI, 0.565–0.889). Detailed reproducibility results are presented in **Supplementary Table S2**.

### Habitat parameter profiles and between-group comparisons

Across the cohort, Hab1 had the lowest ADC (1.173 ± 0.267 × 10^−3^ mm^2^/s) and a relatively low D (0.968 ± 0.279 × 10^−3^ mm^2^/s). Hab2 was characterised primarily by a high f value (0.530 ± 0.114), whereas Hab3 had the highest ADC (1.592 ± 0.688 × 10^−3^ mm^2^/s) and D (1.418 ± 0.713 × 10^−3^ mm^2^/s). These profiles were used to harmonise labels across patients and do not establish microscopic necrosis, oedema, or vascular tissue.

The high-Ki-67 group had lower Hab1 ADC than the low/equal-Ki-67 group (median, 0.947 [0.862–1.161] versus 1.257 [1.113–1.455] × 10^−3^ mm^2^/s; *P* < 0.001; rank-biserial effect size, −0.651) and lower Hab1 D (0.731 [0.650–0.985] versus 1.044 [0.903–1.269] × 10^−3^ mm^2^/s; *P* < 0.001). The Hab3 volume proportion was higher in the high-Ki-67 group (0.396 [0.341–0.488] versus 0.279 [0.190–0.390]; *P* = 0.002), whereas the conventional whole-tumour ADC did not differ significantly (1.236 [1.098–1.503] versus 1.369 [1.197–1.451] × 10^−3^ mm^2^/s; *P* = 0.330).

Within the same BH-corrected eight-feature family, Hab1 f and Hab2 volume proportion were also lower in the high-Ki-67 group (*q* = 0.032 and *q* = 0.006, respectively; **Table 3**; **Figure 3**); neither was retained as a primary imaging predictor. The complete raw *P* values, BH *q* values, and rank-biserial effects are reported in **Table 3**.

**Figure 3:**
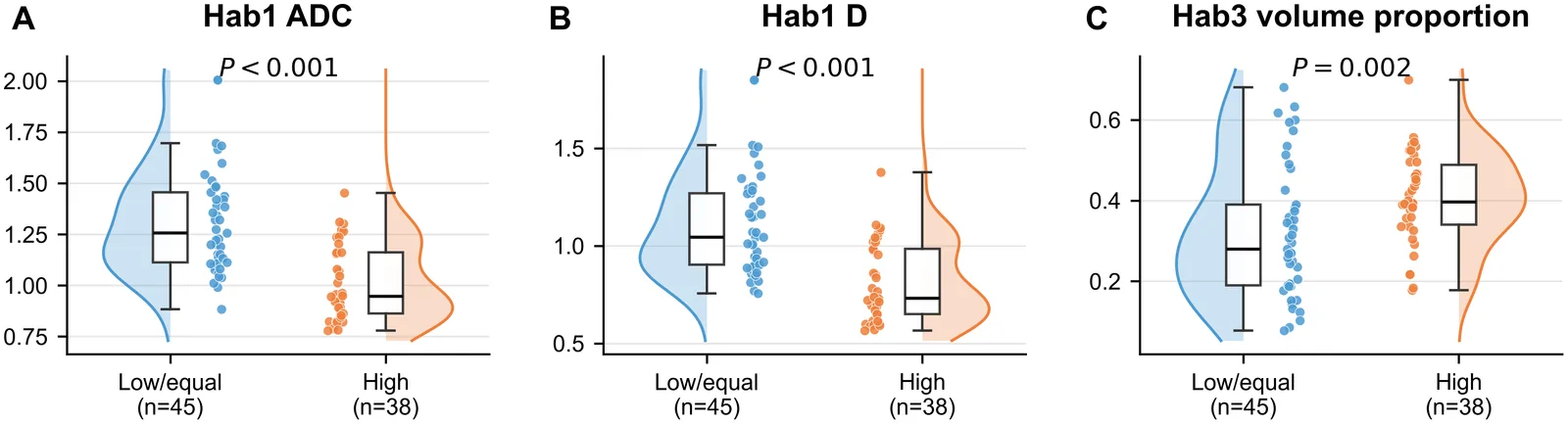
Half-raincloud plots comparing key habitat parameters between high- and low-proliferation groups defined by Ki-67 >70%. **(A)** Apparent diffusion coefficient (ADC) of Habitat 1 (Hab1). **(B)** True diffusion coefficient D of Hab1. **(C)** Volume proportion of Habitat 3 (Hab3). Each panel combines a half-density curve, box plot, and jittered raw data points. The high-proliferation group showed significantly lower Hab1 ADC and Hab1 D values and a significantly higher Hab3 volume proportion.

### Continuous Ki-67 associations and ADC/D redundancy

Continuous Ki-67 was inversely correlated with Hab1 ADC (Spearman *ρ* = −0.502, 95% bootstrap CI, −0.658 to −0.339; *P* < 0.001) and Hab1 D (*ρ* = −0.505, 95% CI, −0.650 to −0.327; *P* < 0.001). The correlation with conventional whole-tumour ADC was weaker (*ρ* = −0.241; *P* = 0.028), whereas the Hab3 volume proportion showed a positive correlation (*ρ* = 0.308, 95% CI, 0.109–0.503; *P* = 0.005). In the multivariable HC3 regression model, the adjusted R^2^ was 0.352 (R^2^ = 0.407; overall *P* < 0.001). Hab1 ADC remained inversely associated with continuous Ki-67 (*β* = −1.99 percentage points per 0.1 × 10^−3^ mm^2^/s; 95% CI, −3.18 to −0.79; *P* = 0.001), whereas the Hab3 volume proportion was not independently associated after adjustment (*β* = 1.70 percentage points per 10 percentage points; 95% CI, −1.42 to 4.81; *P* = 0.286). Poor differentiation and adenocarcinoma status were also associated with continuous Ki-67 in this exploratory model. The corresponding imaging–Ki-67 correlations are visualised in **Figure 4**. Full adjusted coefficients are provided in **Supplementary Table S3**.

**Figure 4:**
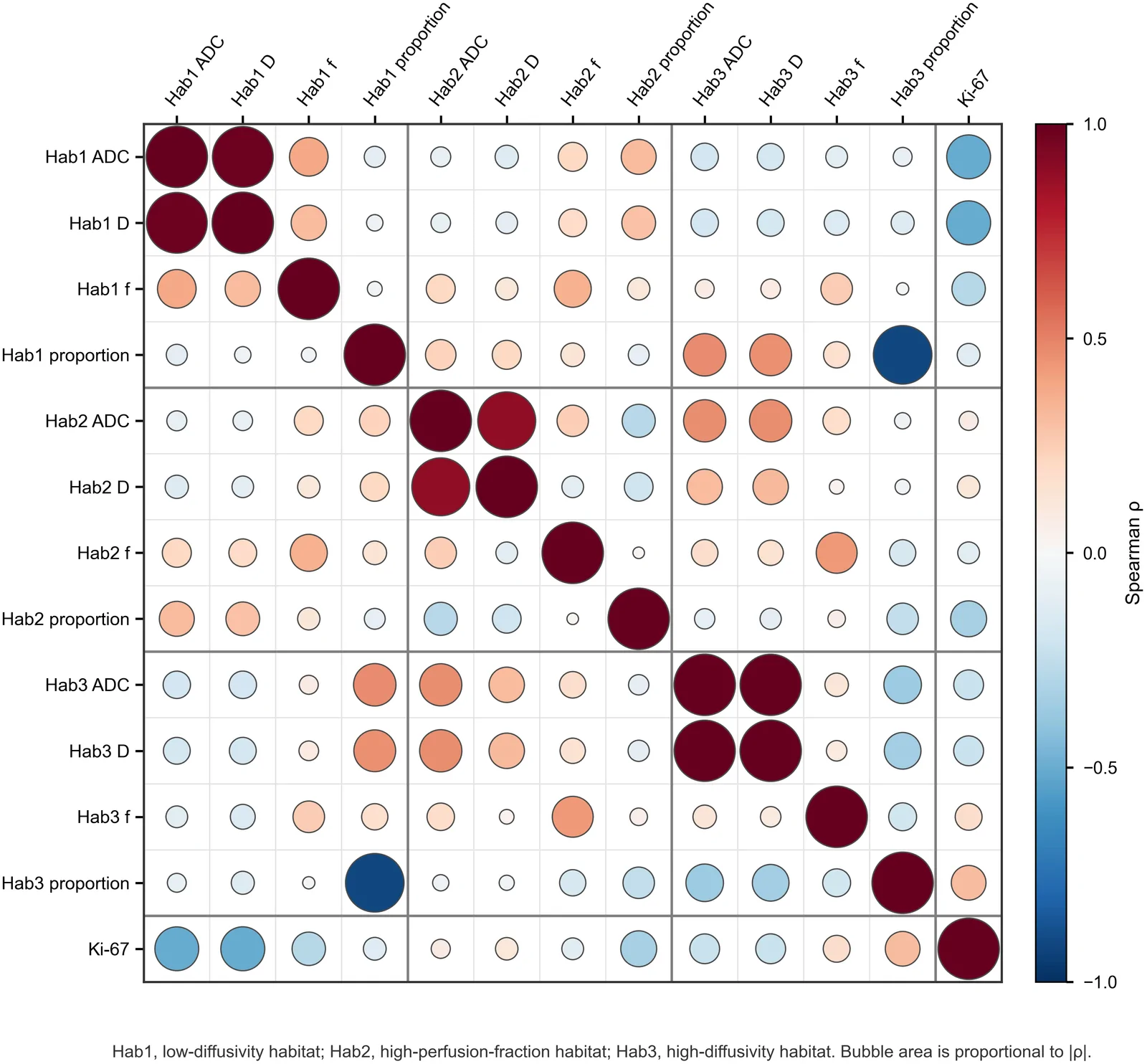
Correlations among imaging-derived habitat metrics and Ki-67. Bubble colour represents the Spearman correlation coefficient (*ρ*), and bubble area is proportional to |*ρ*|. Hab1 denotes the low-diffusivity habitat, Hab2 the high-perfusion-fraction habitat, and Hab3 the high-diffusivity habitat. Volume proportion refers to the fraction of valid tumour voxels assigned to the corresponding habitat.

Hab1 ADC and Hab1 D showed substantial patient-level collinearity (VIF = 30.82 for both). A two-variable PCA of standardised Hab1 ADC and Hab1 D produced a first principal component that remained correlated with continuous Ki-67 (Spearman *ρ* = −0.504; *P* < 0.001). This sensitivity analysis supports the persistence of the diffusion–Ki-67 association after orthogonal transformation. The separately completed voxel-level feature-ablation analysis is reported below.

### Logistic models and apparent discrimination

In the originally reported imaging-only model, lower Hab1 ADC was associated with high Ki-67 status (OR = 0.493 per 0.1 × 10^−3^ mm^2^/s; 95% CI, 0.361–0.672; *P* < 0.001), whereas a higher Hab3 volume proportion was also associated with high Ki-67 status (OR = 1.796 per 10 percentage points; 95% CI, 1.183–2.727; *P* = 0.006). The apparent AUC of Hab1 ADC was 0.826 (95% CI, 0.726–0.909), compared with 0.563 (95% CI, 0.429–0.695) for conventional whole-tumour ADC. The paired DeLong comparison supported a difference between these curves (ΔAUC = 0.263, Z = 3.50, *P* < 0.001).

For the combined imaging model, the fitted probability of high Ki-67 was;


$$p={\left\{1+\exp\left[-\left(5.8235-0.7079\text{X}1+05857\text{X}2\right)\right]\right\}}^{-1},$$

where X1 is Hab1 ADC divided by 0.1 × 10^−3^ mm^2^/s and X2 is the Hab3 volume proportion divided by 0.1. The apparent AUC was 0.877 (95% CI, 0.794–0.938). At the cohort-derived probability threshold of 0.538, sensitivity was 76.3% (95% CI, 62.5%–89.2%), specificity was 84.4% (73.0%–94.7%), positive predictive value was 80.6% (66.7%–92.5%), negative predictive value was 80.9% (68.8%–91.5%), accuracy was 80.7% (72.3%–89.2%), and the F1 score was 0.784; the Brier score was 0.146. For the Hab1 ADC-only model, p ={1+exp[-(7.6162-6.8180A)]}^−1^, where A is Hab1 ADC in units of 10^−3^ mm^2^/s. Its cohort-derived probability threshold of 0.743 corresponded to a Hab1 ADC cut-off of ≤0.962 × 10^−3^ mm^2^/s; at that cut-off, sensitivity was 57.9% (40.6%–72.7%), specificity 97.8% (92.3%–100.0%), positive predictive value 95.7% (86.3%–100.0%), and negative predictive value 73.3% (62.5%–83.1%). All thresholds and operating characteristics are apparent estimates and were not prespecified for clinical use. The combined-model AUC was numerically higher than that of Hab1 ADC alone, but the paired DeLong comparison did not confirm superiority (ΔAUC = 0.051, Z = 1.91, *P* = 0.056; **Figure 5A**).

**Figure 5:**
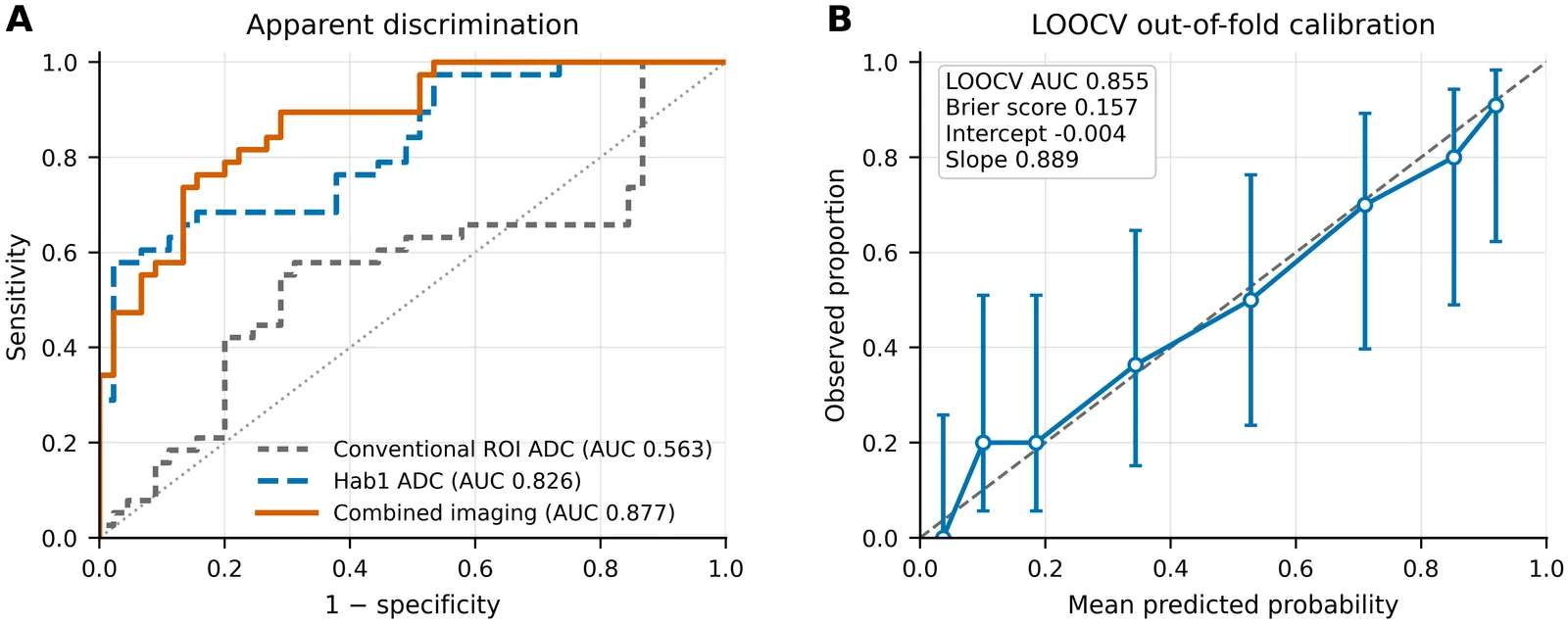
Apparent discrimination and fixed-feature internal calibration of the imaging models. **(A)** Full-cohort receiver operating characteristic curves for conventional ROI ADC, Hab1 ADC, and the combined imaging model comprising Hab1 ADC and the Hab3 volume proportion. The curves and AUCs are apparent, in-sample estimates. **(B)** Calibration of out-of-fold probabilities generated by fixed-feature leave-one-out cross-validation for the combined imaging model. Points show eight equal-frequency probability bins; vertical bars show 95% Wilson confidence intervals for the observed proportions, and the dashed diagonal denotes ideal calibration. The out-of-fold AUC was 0.855 (95% CI, 0.771–0.923), the Brier score was 0.157 (95% CI, 0.112–0.204), the calibration intercept was −0.004 (95% CI, −0.601 to 0.595), and the calibration slope was 0.889 (95% CI, 0.626–1.373). Cross-validation held out patient-level habitat summaries; voxel-level habitat construction was not repeated within each fold.

### Differentiation-adjusted and other sensitivity analyses

Poor differentiation was markedly unevenly distributed between the Ki-67 groups (22/38 in the >70% group versus 3/45 in the ≤70% group). In the fully adjusted Firth model, Hab1 ADC remained inversely associated with high Ki-67 status (OR = 0.554 per 0.1 × 10^−3^ mm^2^/s; 95% CI, 0.381–0.804; *P* = 0.002), whereas the Hab3 volume proportion did not retain independent statistical significance (OR = 1.579 per 0.1 increment; 95% CI, 0.976–2.554; *P* = 0.063). Poor differentiation retained a large but imprecise association (OR = 98.556; 95% CI, 3.765–2,580.056; *P* = 0.006), consistent with sparse cells.

The clinicopathological model yielded a fixed-feature LOOCV AUC of 0.818 (95% CI, 0.721–0.898), compared with 0.854 (0.758–0.927) for the imaging-only model and 0.902 (0.829–0.959) for the combined model. Their corresponding Brier scores were 0.171, 0.156, and 0.128. Adding the fixed imaging pair to the clinicopathological model increased LOOCV AUC by 0.084 (95% bootstrap CI, 0.018–0.159; paired DeLong *P* = 0.016; Holm-adjusted *P* = 0.033) and reduced the Brier score by 0.043 (95% bootstrap CI, 0.003–0.082). Bootstrap optimism-corrected AUCs were 0.843 for the clinicopathological model, 0.870 for the imaging-only model, and 0.916 for the combined model. These findings support internally observed incremental information from the fixed imaging pair, not external clinical utility.

When alternative analytical Ki-67 thresholds were used, the combined imaging model AUC was 0.772 for >60%, 0.877 for >70%, 0.832 for >80%, and 0.772 for ≥70%. These analyses indicate that discrimination depends on the outcome definition and do not establish a clinical Ki-67 threshold. Detailed apparent and fixed-feature LOOCV estimates are provided in **Supplementary Table S3**.

As an additional sensitivity analysis addressing the mixed pathological specimen source, the imaging model was refitted with specimen type (surgical resection versus diagnostic biopsy) added as a fixed covariate. Hab1 ADC remained inversely associated with high Ki-67 status (OR = 0.501 per 0.1 × 10^−3^ mm^2^/s; 95% CI, 0.369–0.681; *P* < 0.001), and Hab3 volume proportion remained positively associated (OR = 1.813 per 0.1 increment; 95% CI, 1.182–2.781; *P* = 0.006). Specimen type itself was not statistically confirmed as an independent predictor in this small cohort (resection versus biopsy: OR = 0.353; 95% CI, 0.106–1.177; *P* = 0.090). The specimen-adjusted fixed-feature LOOCV AUC was 0.858, with a Brier score of 0.152. The specimen variable used in this sensitivity analysis was verified case by case from the original pathology reports. This supportive analysis indicates that the primary imaging associations were not materially changed by adding specimen type, while remaining exploratory because of the retrospective single-centre design and limited sample size. Coefficient and performance details are provided in **Supplementary Table S3**.

### Robustness of habitat construction and downstream prediction

The deterministic one-pixel stress test produced median changes in ROI voxel counts of −25.1% after erosion and +26.5% after dilation. Median ARIs on shared valid voxels were 0.948 and 0.956, respectively, and median semantic agreements were 0.982 and 0.986. Paired changes in Hab1 ADC and the Hab3 volume proportion were small and were not significant after Holm correction. The matched-cohort LOOCV AUC decreased from 0.852 to 0.775 after erosion (ΔAUC = −0.077; paired-bootstrap 95% CI, −0.145 to −0.016; Holm-adjusted DeLong *P* = 0.046), whereas the change after dilation was not statistically confirmed (ΔAUC = −0.039; 95% CI, −0.102 to 0.005; adjusted *P* = 0.152). In the complementary ± 5% and ± 10% volume-matched analyses, median ARIs were 0.977–0.987 and semantic agreements were 0.993–0.996. Hab1 ADC shifted by less than 0.3% of its baseline median, the Hab3 volume proportion showed no Holm-adjusted difference, and LOOCV AUC changes ranged from −0.011 to −0.002; all paired-bootstrap intervals included zero, and all Holm-adjusted DeLong *P* values were ≥0.432.

K-means initialisation was numerically stable. Across 20 alternative random states per patient, the cohort median of the patient-specific worst-run ARI was 0.993 (95% bootstrap CI, 0.991–0.997), and the corresponding worst-run semantic agreement was 0.998 (95% CI, 0.997–0.999). The cohort medians of the maximum absolute changes were 0.00079 × 10^−3^ mm^2^/s for Hab1 ADC and 0.000915 for the Hab3 volume proportion. The seed-42 model had an LOOCV AUC of 0.855; across the 20 alternative seeds, AUCs ranged from 0.851 to 0.856 (median, 0.854), with maximum absolute change of 0.004 and no significant paired DeLong comparison after Holm correction. The patient-level median of the maximum absolute change in LOOCV probability was 0.008, and 78 of 83 patients had a maximum change ≤0.05.

With patient-level z-score scaling, median ARIs relative to the locked D+ADC+f representation were 0.847 for ADC+f, 0.865 for D+f, and 0.983 for diffusion-PC1+f; their LOOCV AUCs were 0.827, 0.726, and 0.852, respectively, compared with 0.855 for the locked representation. Holding D+ADC+f fixed, patient-level median/interquartile-range scaling, cohort-level z-score scaling, and no scaling yielded median ARIs of 0.918, 0.955, and 0.389 and LOOCV AUCs of 0.775, 0.791, and 0.581, respectively. Thus, the orthogonalised diffusion representation closely reproduced the locked result, whereas omission of scaling materially changed the partition and model performance.

Patient-level silhouette analysis also supported *k* = 3 as the fixed working solution: the cohort median was 0.460 at *k* = 3, compared with 0.435, 0.413, and 0.409 at *k* = 2, *k* = 4, and *k* = 5, respectively. The overall paired difference was significant (Friedman χ^2^ (3) = 63.101, *P* = 1.28×10^−13^), and all three *k* = 3 contrasts remained significant after Holm correction (all *P* ≤ 0.00018). LOOCV AUC was 0.855 at *k* = 3, compared with 0.791 for the exploratory *k* = 2 surrogate and 0.772 and 0.777 after outcome-blind coarsening of *k* = 4 and *k* = 5, respectively. These alternative-*k* analyses were structural sensitivity tests and were not interpreted as defining interchangeable biological habitats. Separately from this voxel-level cluster-number sensitivity analysis, the secondary cohort-level patient-phenotype analysis is visualised in **Figure 2**; it was outcome-blind and was not used to define, train, or validate the primary Ki-67 model.

### Internal validation and permutation test

Fixed-feature leave-one-out validation of the combined imaging model yielded an out-of-fold AUC of 0.855 (95% CI, 0.771–0.923), a Brier score of 0.157 (95% CI, 0.112–0.204), a calibration intercept of −0.004 (95% CI, −0.601 to 0.595), and a calibration slope of 0.889 (95% CI, 0.626–1.373; **Figure 5B**). The corresponding out-of-fold AUC for Hab1 ADC alone was 0.816. Bootstrap optimism correction estimated an AUC of 0.871 for the combined model and 0.828 for Hab1 ADC alone. These procedures resampled or held out patient-level summary features; the voxel-level fitting, ROI construction, label harmonisation, and cluster-number choice were not repeated inside each held-out fold. Instead, the locked voxel-level pipeline was evaluated separately with outcome-blind boundary, initialisation, feature, scaling, and cluster-number sensitivity analyses. In the 1,000-refit label-permutation test, none of the valid permutations reached the observed combined-model AUC, giving a corrected permutation *P* = 0.001. The primary apparent and fixed-feature model estimates are summarised in **Table 4**, and representative parameter maps and image-defined habitats are shown in **Figure 6**.

**Table 4:** Apparent and fixed-feature internal validation performance of the primary imaging models.

Model	Apparent AUC (95% CI)	Apparent Brier score	Fixed-feature LOOCV AUC (95% CI)	LOOCV Brier score (95% CI)	LOOCV calibration
Conventional ROI ADC	0.563 (0.429–0.695)	0.241	—	—	—
Hab1 ADC	0.826 (0.726–0.909)	0.167	0.816*	—	—
Hab1 ADC + Hab3 volume proportion	0.877 (0.794–0.938)	0.146	0.855 (0.771–0.923)	0.157 (0.112–0.204)	Intercept −0.004 Slope 0.889

Apparent values were estimated in the full cohort and are not validation results. Fixed-feature LOOCV held out patient-level summary features; the voxel-level habitat-construction pipeline was not rerun within each fold. *The out-of-fold AUC for Hab1 ADC alone was reported in the revised analysis, but a separate bootstrap confidence interval, Brier score, and calibration estimates were not separately tabulated. AUC, area under the receiver operating characteristic curve; CI, confidence interval; LOOCV, leave-one-out cross-validation; ROI, region of interest.

**Figure 6:**
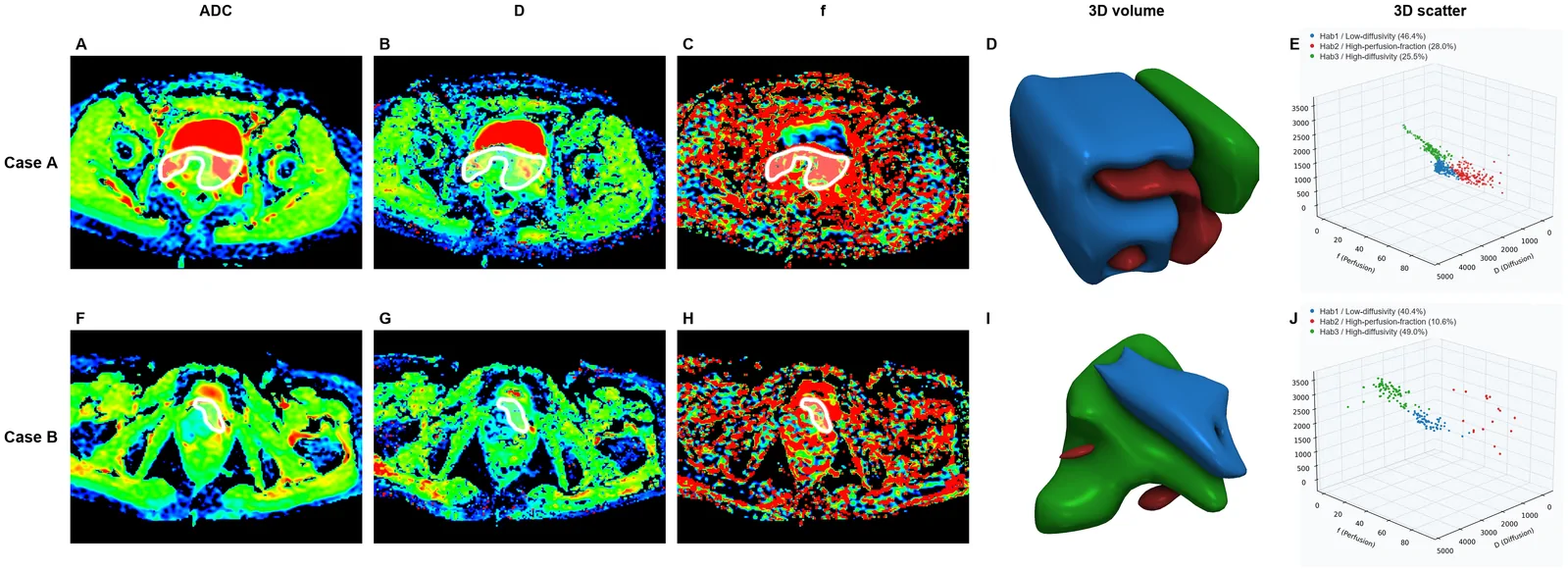
Representative IVIM parameter maps and image-defined habitats in lower- and higher-Ki-67 cervical squamous cell carcinoma. Case A was a 62-year-old patient with Ki-67 of 60%; Case B was a 58-year-old patient with Ki-67 of 90%. **(A–C, F–H)** show ADC, D, and perfusion-fraction (f) maps on the axial slice containing the largest tumour ROI area. Semi-transparent shaded regions with white contours indicate the tumour ROI used for habitat analysis. The maps are retained as qualitative representative displays. **(D, I)** show the three-dimensional spatial distribution of the image-defined habitats; **(E, J)** show their voxel distributions in D-f-ADC feature space. Habitat volume proportions were Hab1/Hab2/Hab3 = 46.4%/28.0%/25.5% in Case A and 40.4%/10.6%/49.0% in Case (B) Hab1, Hab2, and Hab3 denote the locked low-diffusivity, high-perfusion-fraction, and high-diffusivity imaging-profile classes, respectively; they are not histologically validated tissue identities.

## Discussion

The main finding was that ADC measured within the low-diffusivity habitat was more strongly related to Ki-67 than conventional whole-tumour ADC in this single-centre cohort. Hab1 ADC showed a moderate inverse continuous association with Ki-67 and an apparent AUC of 0.826, whereas conventional ROI ADC had an AUC of 0.563. This direction agrees with prior cervical-cancer IVIM work in which lower D was observed in the higher-Ki-67 group (9). It is also compatible with recent DWI, non-Gaussian diffusion, and ADC-histogram studies linking restricted diffusion or distributional descriptors to Ki-67 and tumour-microenvironment features (10, 11, 25). Longitudinal functional imaging has shown that heterogeneity estimates can differ across imaging modalities and time points (26). Together, these observations support testing localised diffusion summaries rather than assuming that a single whole-tumour average captures all relevant variation. They do not, however, establish that Hab1 is histologically pure or that diffusion restriction is caused by proliferation alone.

Adding the Hab3 volume proportion increased the apparent AUC from 0.826 to 0.877, but the paired DeLong comparison gave *P* = 0.056, and Hab3 was not independently associated with continuous Ki-67 after adjustment. The combined-model result should therefore be described as a numerical improvement rather than statistically established superiority or proven incremental clinical value. This cautious interpretation is consistent with cervical habitat studies in which multiparametric subregions improved prediction of lymphovascular-space invasion or treatment response, but performance depended on the selected imaging inputs, cohort, and validation design (13, 14). More recent prospective and dual-centre studies illustrate the potential of habitat approaches while also emphasizing the importance of independent testing (15, 16). In the present study, Hab3 should be regarded as a potentially complementary feature, not a confirmed independent predictor.

Histological differentiation is an important interpretive constraint. Poorly differentiated tumours accounted for 22 of 38 high-Ki-67 cases but only 3 of 45 low/equal-Ki-67 cases. The persistence of the Hab1 ADC association after full Firth adjustment argues against differentiation being the sole explanation for the imaging finding. Moreover, the combined model showed better fixed-feature LOOCV discrimination and lower overall prediction error than the fixed-covariate clinicopathological model (ΔAUC = 0.084 and Brier improvement = 0.043), supporting complementary information from the fixed imaging pair within this cohort. Ki-67 has previously been associated with clinicopathological characteristics and prognosis in cervical cancer (3), but that literature does not establish a causal relation between differentiation, proliferation, and the observed imaging phenotype. The full model also remains parameter-rich for 83 patients, several pathological strata are sparse, and external validation is unavailable; the results therefore support an internally observed association rather than established clinical utility.

The revised terminology deliberately limits biological interpretation. IVIM biomarkers are model-derived and can vary with acquisition, fitting strategy, and model adequacy (6, 8, 17). Hab3 was defined by its higher D/ADC profile and is therefore termed a high-diffusivity imaging habitat; Hab2 is termed a high-perfusion-fraction habitat because it had the highest mean f. These parameter profiles are not direct diagnoses of microscopic necrosis, oedema, stromal looseness, vascular abundance, or angiogenesis. Similarly, the lower diffusion metrics of Hab1 may be compatible with greater diffusion restriction, but a direct link to cellularity remains a hypothesis. Current habitat-imaging methodology treats such partitions as image-derived phenotypes and identifies spatial pathology matching as a necessary step for biological validation (12). Because gross necrosis was excluded during ROI delineation and imaging voxels were not spatially registered to histopathological sections, stronger tissue-specific labels are not justified here.

Patient-specific standardisation improved within-patient clustering but creates a cross-patient comparability challenge. K-means cluster indices have no intrinsic ordering, and their interpretation depends on the feature representation and initialisation (18, 19). In the locked workflow, arbitrary indices were not treated as biological labels; clusters were harmonised using predefined parameter-profile rules. Even so, because clustering was performed separately for each patient and spatial coordinates were not included, the classes should be regarded as phenotype-defined voxel groups rather than universally comparable anatomical habitats. This distinction is particularly important when comparing features across institutions or implementing a locked model.

The robustness analyses indicate that the main findings were not produced by one favourable random initialisation or by minor contour variation. Repeated K-means initialisation produced nearly identical partitions, features, and model AUCs, while ±5% and ±10% volume-matched boundary changes had little effect on cohort-level discrimination. The one-pixel erosion stress test was less reassuring: although habitat summaries remained similar, model AUC decreased after removal of a median of one-quarter of ROI voxels. This should be interpreted as a stringent stress test, not as representative repeat-contouring variability. The residual sensitivity accords with broader concerns about quantitative IVIM repeatability (17) and habitat-workflow standardisation (12). Thus, the data support stability under mild perturbation but do not demonstrate segmentation invariance in every patient.

The representation analyses also clarify why patient-level z-score scaling and k = 3 were retained. The diffusion-PC1+f representation closely reproduced the locked D+ADC+f result, indicating that the association was not created solely by double-weighting the correlated D and ADC variables. Omitting scaling substantially changed both partitions and discrimination, as expected when Euclidean distance is applied to variables with different distributions (19). Patient-level scaling was retained because it equalises feature contributions within each tumour without estimating a transform from the full cohort. Likewise, k = 3 had the highest cohort-level patient-specific silhouette and was selected without reference to Ki-67. These findings make the analytical choices empirically defensible within this cohort, but do not establish patient-level z-scoring or k = 3 as universally optimal.

The continuous-outcome and threshold analyses support a less threshold-dependent narrative. Hab1 ADC remained associated with continuous Ki-67 after adjustment, whereas discrimination changed when the binary threshold was moved from >60% to >80%. A previous cervical IVIM study used a 50% Ki-67 threshold (9), illustrating that published definitions are study-specific rather than interchangeable clinical standards. The >70% definition in the present study should therefore be treated as a cohort-level analytical definition. Leave-one-out validation and permutation testing provide internal support for the fixed-feature patient-level model, but neither establishes transportability across institutions, scanners, acquisition protocols, or pathological workflows.

This study did not evaluate clinical utility. High Ki-67 status was not linked in the protocol to a specific treatment decision, biopsy strategy, surgical plan, or follow-up action, and neither a probability threshold nor the relative consequences of false-positive and false-negative predictions was prespecified. Accordingly, no net benefit or clinical actionability claim is made. A future prospective study should define the downstream action and acceptable prediction-error trade-off before evaluating a locked model at prespecified thresholds. Multicentre cervical habitat studies demonstrate the feasibility of independent-site assessment (13, 16). Two-centre PET and MRI nodal habitat studies also provide examples of cross-site or risk-stratification evaluation (27, 28), but a comparable external cohort is not available for the present model.

The fitted equations are supplied for transparent reporting and third-party evaluation, not deployment. Their current use requires expert tumour ROI delineation, valid multi-b-value IVIM inputs, and execution of the locked preprocessing and habitat scripts; the models do not define a safe procedure for missing or non-convergent inputs. Cases with insufficient valid voxels or unreliable images must not be assigned a model probability. A deployable tool would require automated quality control, external testing, and a prespecified user workflow.

Several limitations should be considered. First, this was a retrospective single-centre study of 83 patients without external validation. The dataset did not retain ethnicity, socioeconomic status, or other variables required for a meaningful fairness assessment, and reliable subgroup-performance estimation was not possible. Second, eligibility required baseline multi-b-value IVIM-DWI before biopsy sampling, lesion excision, or chemoradiotherapy and an available pathological Ki-67 result. Although patients were screened chronologically from the gynaecology outpatient source population and all eligible analysable cases were retained, these restrictions may limit generalisability. Third, fixed patient-level habitat summaries were used during model resampling; voxel-level reconstruction was tested in separate sensitivity analyses rather than repeated within every resampling split. Performance remained dependent on feature scaling, cluster number, and the more severe erosion stress test. Fourth, although acquisition and fitting settings were recovered from DICOM headers, converted sidecars, and source code, the analysis did not use a residual-based convergence threshold, explicit magnitude-noise correction, or additional susceptibility-distortion correction. Finally, pathological Ki-67 was based on hotspot counting in biopsy material from 49 patients or surgical material from 34 patients and was not spatially registered to the imaging habitats. Specimen source and MRI-to-pathology timing were verified case by case; the overall median interval was 1 day [Q1–Q3, 0–3 days], and the range was 0–7 days. Adjustment for specimen type did not materially change the imaging associations, but combining biopsy and resection material may still introduce sampling heterogeneity, and no independent pathology rescore was available to quantify Ki-67 reader reproducibility. These limitations define the study as an exploratory proof of concept and motivate prospective multicentre validation with a locked pipeline and spatially matched pathological assessment.

## Conclusion

In this retrospective single-centre cohort, the low-diffusivity IVIM habitat ADC showed a stronger association with Ki-67 status than conventional whole-tumour ADC. The combined model of low-diffusivity ADC and high-diffusivity habitat volume proportion model had numerically higher apparent discrimination, but its superiority over low-diffusivity ADC alone was not statistically confirmed (paired DeLong *P* = 0.056). The findings support further evaluation of IVIM habitat features as exploratory whole-tumour imaging biomarkers, while external validation, pathology-linked spatial validation, and prospective confirmation of the locked normalisation, cluster-number, and segmentation procedures remain necessary before clinical translation.

## Data Availability

The raw data supporting the conclusions of this article will be made available by the authors, without undue reservation.
