# Supplementary material for: IVIM-based habitat imaging for non-invasive assessment of Ki-67 proliferation status in cervical cancer
Source: Front Oncol. 2026 Oct 14;16:1885498. doi: 10.3389/fonc.2026.1885498 (PMC13713370; doi:10.3389/fonc.2026.1885498)
Supplement: Supplementary file 2 [file DataSheet2.pdf]

CLAIM 2024 checklist — completed

Scope: CLAIM 2024 was not formally extended to imaging-biomarker/radiomics research; it is applied conservatively by analogy because the reviewer requested it. Page numbers refer to Manuscript_26.09.13_R2_FINAL. A checked Yes means the item is explicitly addressed, including a transparently reported limitation; N/A denotes a genuinely inapplicable item.

Section	Item	CLAIM 2024 checklist item	Yes	No	N/A	Page(s) / explanation
Title/Abstract	1	Identify the AI/ML method in the title or abstract	X			1–2; title and Abstract identify K-means habitat modelling.
Title/Abstract	2	Structured summary of design, data, methods, results, conclusions, and availability	X			2; structured Abstract. Data/code availability is reported on p. 16.
Introduction	3	Scientific/clinical background and intended role	X			2–3, 13–15; exploratory stratification, not deployment.
Introduction	4	Aims, objectives, and hypotheses	X			3; final Introduction paragraph.
Methods	5	Prospective or retrospective study design	X			2–4; retrospective single-centre cohort.
Methods	6	Study goal and prediction target	X			2–3, 6–8; Ki-67 >70% with continuous/threshold sensitivity analyses.
Methods	7	Data sources and alignment with the target population	X			3–4, 15; hospital cohort and representativeness limitation.
Methods	8	Inclusion, exclusion, and case-identification procedures	X			3–4, 24; chronological screening and complete flow.
Methods	9	Data preprocessing and quality control	X			4–6; conversion, b-value checks, native-grid processing, QC, and scaling.
Methods	10	Selection, cropping, or annotation of image data	X			5; manual solid-tumour ROI/VOI procedure and exclusions.
Methods	11	De-identification of data	X			3, 16; retrospective de-identified data reported at policy level.
Methods	12	Handling of missing data	X			7; no missing values in analysed variables and no imputation.
Methods	13	Image-acquisition protocol	X			4; scanner/sequence parameters; Supplementary Table S1.
Methods	14	Reference-standard method	X			6–7; Ki-67 assay, scoring, specimen source, timing, and blinding.
Methods	15	Rationale and limitations of the reference standard	X			6–7, 15; threshold sensitivity and sampling limitations.
Methods	16	Source and qualifications of reference-standard assessors	X			7; senior pathologist and mutual blinding.
Methods	17	Annotation/reference-standard procedure for evaluated cases	X			6–7; hotspot/500-cell procedure and timing reported.
Methods	18	Inter- and intrarater variability	X			5, 7, 15; imaging ICCs reported; no independent Ki-67 rescore is disclosed.
Methods	19	Assignment to development, tuning, and test partitions	X			8; no fixed split; patient-level fixed-feature LOOCV.
Methods	20	Level at which data partitions are disjoint	X			7–9; entire patients held out, one row per patient.
Methods	21	Intended sample size and rationale	X			4; all eligible/QC-passing cases retained; no a priori calculation.
Methods	22	Detailed model description	X			5–9, 12; equations/settings and Supplementary Table S8.
Methods	23	Software, libraries, and versions	X			4–9; retained versions reported; unavailable PyTorch patch disclosed.
Methods	24	Parameter initialisation	X			5–6; IVIM initial values and K-means++/seed settings.
Methods	25	Training approach, hyperparameters, and stopping criteria	X			5–9; applicable IVIM/K-means/regression settings reported.
Methods	26	Selection of the final model	X			5–6, 8; outcome-blind k selection and a priori predictors.

Section	Item	CLAIM 2024 checklist item	Yes	No	N/A	Page(s) / explanation
Methods	**27**	Ensembling methods			**X**	N/A; no ensemble model was used.
Methods	**28**	Performance metrics	**X**			8–12; discrimination, calibration, Brier, and operating characteristics.
Methods	**29**	Statistical uncertainty, significance, and analysis software	**X**			7–12; CIs, DeLong/bootstrap, q values, and software reported.
Methods	**30**	Robustness and sensitivity analyses	**X**			6, 9–12; ROI, k/seed/scaling, ablation, threshold, and permutation analyses.
Methods	**31**	Explainability or interpretability methods			**X**	N/A as a separate post-hoc method; low-dimensional equations are directly reported.
Methods	**32**	Evaluation on internal data	**X**			8, 10–12; fixed-feature LOOCV, calibration, bootstrap optimism, permutation.
Methods	**33**	External testing			**X**	N/A; no external cohort. This limitation is explicit on p. 15.
Methods	**34**	Clinical-trial registration			**X**	17; Registration: Not applicable; this was a retrospective observational study.
Results	**35**	Numbers included and excluded	**X**			2–4, 9, 24; 149 → 100 → 83 with reasons.
Results	**36**	Demographic/clinical characteristics and potential bias	**X**			9, 15, 20; available characteristics and missing fairness variables disclosed.
Results	**37**	Final performance and comparator/reference results	**X**			10–12, 23, 26; imaging, conventional ROI, clinical, and combined models.
Results	**38**	Classification accuracy, precision, ROC, and calibration	**X**			10–12, 23, 26; estimates and CIs reported.
Results	**39**	Failure analysis or confusion matrix	**X**			12 and Supplementary Table S8; aggregate TN/FP/FN/TP reported.
Discussion	**40**	Study limitations	**X**			15; design, sample, validation, pathology, and transportability limitations.
Discussion	**41**	Implications and intended clinical role	**X**			13–15; no current utility/deployment claim.
Other	**42**	Protocol or additional technical details	**X**			17; Protocol: No separate study protocol was published; technical methods are on pp. 3–9 and in Supplements.
Other	**43**	Software, model, and data availability	**X**			16; privacy/DSA conditions and code/source-table availability reported.
Other	**44**	Funding source and role	**X**			17; Role of funder: the funders had no role in study design, data collection, analysis, interpretation, manuscript preparation, or the decision to submit.

Reporting boundaries: no independent Ki-67 rescore or external cohort was available; excluded cases lacked a complete standardised covariate set; the revised manuscript explicitly reports the confirmed protocol, registration, patient/public involvement, and funder-role statements in the Declarations; no external cohort or independent Ki-67 rescore was available.
