# Supplementary material for: IVIM-based habitat imaging for non-invasive assessment of Ki-67 proliferation status in cervical cancer
Source: Front Oncol. 2026 Oct 14;16:1885498. doi: 10.3389/fonc.2026.1885498 (PMC13713370; doi:10.3389/fonc.2026.1885498)
Supplement: Supplementary file 3 [file DataSheet3.pdf]

STROBE Statement—checklist of items that should be included in reports of observational studies

	Item No.	Recommendation	Page No.	Relevant text from manuscript
**Title and abstract**	1	(*a*) Indicate the study's design with a commonly used term in the title or the abstract	1–2	Title; structured Abstract identifies the retrospective cohort design.
		(*b*) Provide in the abstract an informative and balanced summary of what was done and what was found	2	Abstract summarises objective, cohort, methods, principal results, and limitations.
**Introduction**				
Background/rationale	2	Explain the scientific background and rationale for the investigation being reported	2–3	Introduction—clinical background, IVIM rationale, and evidence gap.
Objectives	3	State specific objectives, including any prespecified hypotheses	3	Introduction—prespecified study objectives and exploratory prediction aim.
**Methods**				
Study design	4	Present key elements of study design early in the paper	3	Methods—retrospective single-centre observational cohort.
Setting	5	Describe the setting, locations, and relevant dates, including periods of recruitment, exposure, follow-up, and data collection	3–4	Setting, recruitment period (Jan 2019–Jun 2025), and data collection described.
Participants	6	(*a*) *Cohort study*—Give the eligibility criteria, and the sources and methods of selection of participants. Describe methods of follow-up<br>*Case-control study*—Give the eligibility criteria, and the sources and methods of case ascertainment and control selection. Give the rationale for the choice of cases and controls<br>*Cross-sectional study*—Give the eligibility criteria, and the sources and methods of selection of participants	3–4	Eligibility, chronological screening, participant source, and exclusions; no follow-up endpoint.
		(*b*) *Cohort study*—For matched studies, give matching criteria and number of exposed and unexposed<br>*Case-control study*—For matched studies, give matching criteria and the number of controls per case	N/A	Unmatched cohort; no exposed/unexposed matching was used.
Variables	7	Clearly define all outcomes, exposures, predictors, potential confounders, and effect modifiers. Give diagnostic criteria, if applicable	5–8	Ki-67 outcome, imaging predictors, clinicopathological covariates, and definitions.
Data sources/ measurement	8*	For each variable of interest, give sources of data and details of methods of assessment (measurement). Describe comparability of assessment methods if there is more than one group	4–7	MRI acquisition, preprocessing, ROI/VOI, habitat features, and pathology assessment.
Bias	9	Describe any efforts to address potential sources of bias	5–9, 15	Blinding, QC, robust regression, sensitivity analyses, and limitations address bias.
Study size	10	Explain how the study size was arrived at	4	All chronologically screened eligible/QC-passing cases retained; no a priori calculation.

Quantitative variables	11	Explain how quantitative variables were handled in the analyses. If applicable, describe which groupings were chosen and why	7–9	Scaling, coding, continuous Ki-67 analysis, and prespecified >70% threshold reported.
Statistical methods	12	(*a*) Describe all statistical methods, including those used to control for confounding	7–9	Statistical tests, models, validation, uncertainty, and multiplicity control described.
		(*b*) Describe any methods used to examine subgroups and interactions	7, 15	Subgroup/fairness analyses were infeasible; available sensitivity analyses reported.
		(*c*) Explain how missing data were addressed	7	No missing values in analysed variables; no imputation.
		(*d*) *Cohort study*—If applicable, explain how loss to follow-up was addressed<br>*Case-control study*—If applicable, explain how matching of cases and controls was addressed<br>*Cross-sectional study*—If applicable, describe analytical methods taking account of sampling strategy	N/A	No longitudinal follow-up, matching, or complex survey sampling.
		(*e*) Describe any sensitivity analyses	6, 9–12	ROI perturbation, k/seed/scaling/ablation, threshold, pathology, bootstrap, and permutation analyses.
**Results**				
Participants	13*	(a) Report numbers of individuals at each stage of study—eg numbers potentially eligible, examined for eligibility, confirmed eligible, included in the study, completing follow-up, and analysed	2–4, 9, 24	149 screened, 100 eligible, 83 analysed; outcome counts and flow diagram reported.
		(b) Give reasons for non-participation at each stage	3–4, 24	All exclusion reasons and counts are itemised in Methods and Figure 1.
		(c) Consider use of a flow diagram	24	Figure 1 provides the participant-flow diagram.
Descriptive data	14*	(a) Give characteristics of study participants (eg demographic, clinical, social) and information on exposures and potential confounders	9, 20	Participant demographic and clinicopathological characteristics are in Results/Table 1.
		(b) Indicate number of participants with missing data for each variable of interest	7	No missing values in the analysed variables.
		(c) *Cohort study*—Summarise follow-up time (eg, average and total amount)	N/A	No longitudinal follow-up outcome; MRI-to-pathology timing is reported on p. 7.
Outcome data	15*	*Cohort study*—Report numbers of outcome events or summary measures over time	2, 9	High Ki-67 n=38; Ki-67 ≤70% n=45.
		Case-control study—Report numbers in each exposure category, or summary measures of exposure	N/A	Not a case-control analysis.
		Cross-sectional study—Report numbers of outcome events or summary measures	N/A	Not a cross-sectional analysis.
Main results	16	(*a*) Give unadjusted estimates and, if applicable, confounder-adjusted estimates and their precision (eg, 95% confidence interval). Make clear which confounders were adjusted for and why they were included	9–12, 22–23	Effect estimates, 95% CIs, model coefficients, and performance comparisons reported.
		(*b*) Report category boundaries when continuous variables were categorized	7	High Ki-67 defined as >70%; sensitivity thresholds also specified.
		(*c*) If relevant, consider translating estimates of relative risk into absolute risk for a meaningful time period	N/A	No time-to-event relative-risk analysis.
Other analyses	17	Report other analyses done—eg analyses of subgroups and interactions, and sensitivity analyses	9–12	Robustness, sensitivity, ablation, continuous-outcome, and comparator analyses reported.
**Discussion**				

Key results	18	Summarise key results with reference to study objectives	13	Discussion opens with the key findings in relation to the objectives.
Limitations	19	Discuss limitations of the study, taking into account sources of potential bias or imprecision. Discuss both direction and magnitude of any potential bias	15	Limitations address selection, sample size, internal validation, pathology, and generalisability.
Interpretation	20	Give a cautious overall interpretation of results considering objectives, limitations, multiplicity of analyses, results from similar studies, and other relevant evidence	13–15	Cautious interpretation integrates aims, uncertainty, limitations, and prior evidence.
Generalisability	21	Discuss the generalisability (external validity) of the study results	15	Single-centre transportability and need for multicentre external validation discussed.
**Other information**				
Funding	22	Give the source of funding and the role of the funders for the present study and, if applicable, for the original study on which the present article is based	17	Funding sources/grant numbers and the confirmed Role of funder statement are reported in Declarations.

*Give information separately for cases and controls in case-control studies and, if applicable, for exposed and unexposed groups in cohort and cross-sectional studies.

Note: An Explanation and Elaboration article discusses each checklist item and gives methodological background and published examples of transparent reporting. The STROBE checklist is best used in conjunction with this article (freely available on the Web sites of PLoS Medicine at http://www.plosmedicine.org/, Annals of Internal Medicine at http://www.annals.org/, and Epidemiology at http://www.epidem.com/). Information on the STROBE Initiative is available at www.strobe-statement.org.
