# Supplementary material for: IVIM-based habitat imaging for non-invasive assessment of Ki-67 proliferation status in cervical cancer
Source: Front Oncol. 2026 Oct 14;16:1885498. doi: 10.3389/fonc.2026.1885498 (PMC13713370; doi:10.3389/fonc.2026.1885498)
Supplement: Supplementary file 4 [file DataSheet4.pdf]

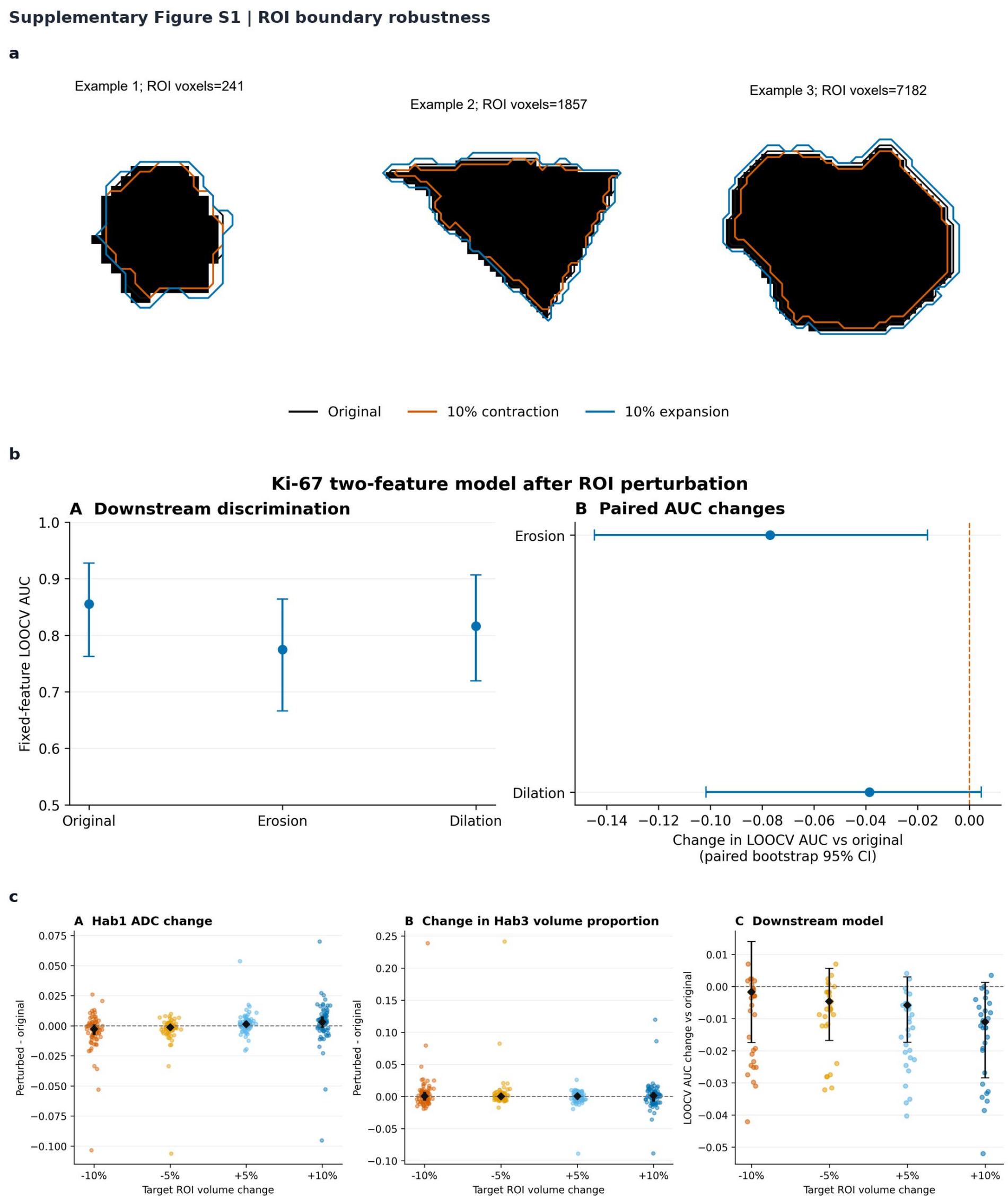

Supplementary Figure S1. ROI boundary robustness. (a) Representative volume-matched boundary perturbations. The examples correspond approximately to the 10th, 50th, and 90th percentiles of original ROI voxel count and show the original contour together with one 10% contraction and one 10% expansion. Perturbations remained on the current in-plane boundary and preserved the originally occupied axial slices. (b) Downstream model sensitivity in the deterministic one-pixel in-plane erosion/dilation stress test. Internal panel A shows fixed-feature leave-one-out cross-validation (LOOCV) AUC estimates with patient-bootstrap 95% confidence intervals; internal panel B shows paired AUC changes relative to the matched original ROI with paired-bootstrap 95% confidence intervals. Erosion was evaluable in 82 patients and dilation in 83 patients. The vertical dashed line denotes no AUC change. (c) Mild proportional boundary sensitivity at exact ROI volume changes of −10%, −5%, +5%, and +10%. Internal panels A and B show patient-level median changes in Hab1 ADC and Hab3 volume proportion across 30 fixed Monte Carlo realizations per patient and condition. Internal panel C shows Monte Carlo realization-specific model estimates together with the model based on patient-median features (black diamonds and paired patient-bootstrap 95% confidence intervals). These analyses assess internal boundary sensitivity and do not establish segmentation invariance.

Supplementary Figure S2 | K-means initialization stability

a

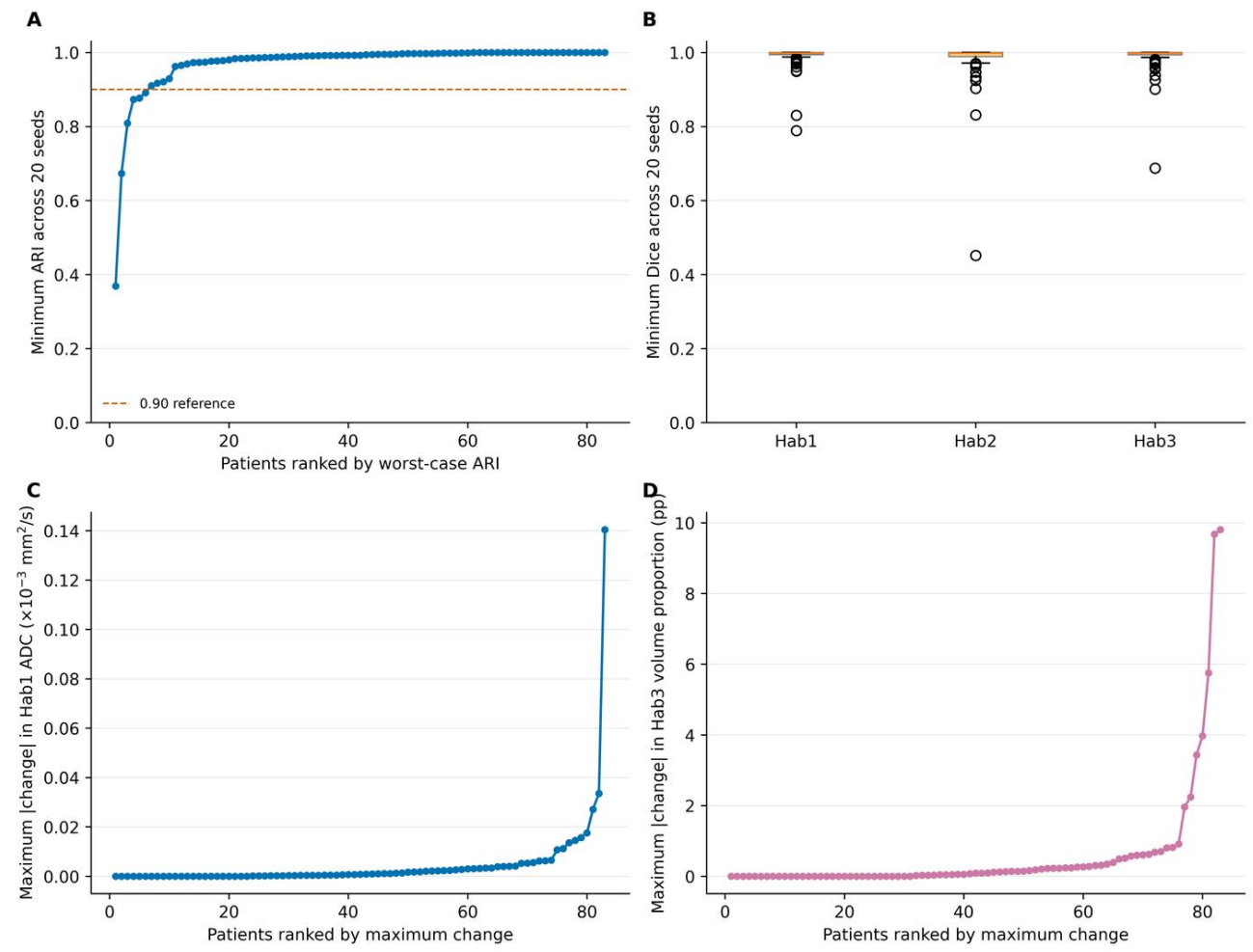

b

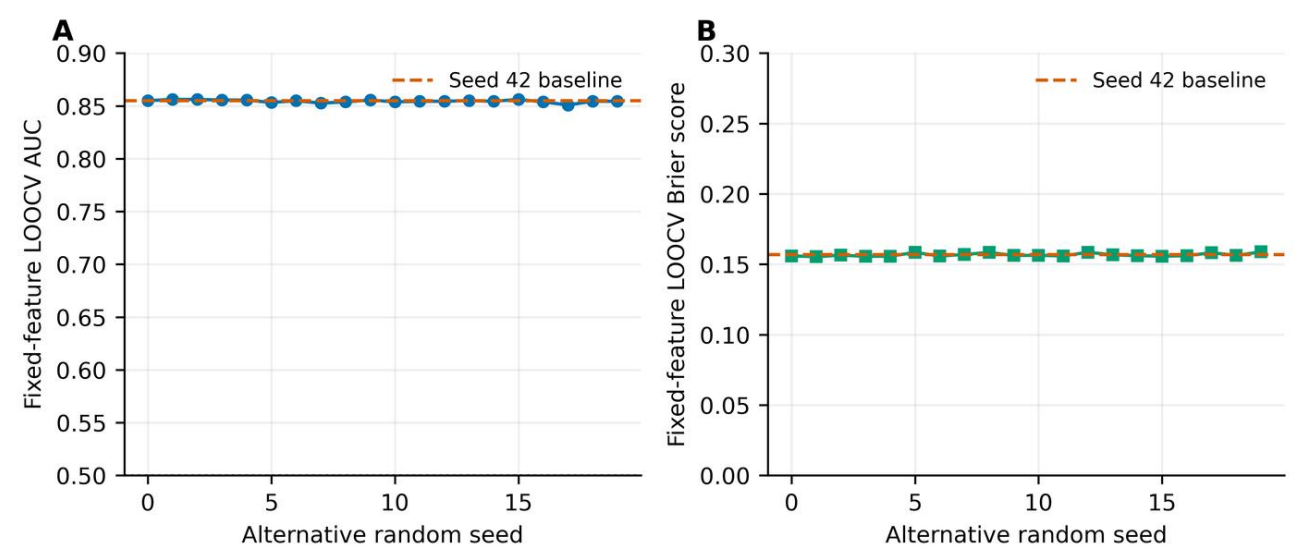

c

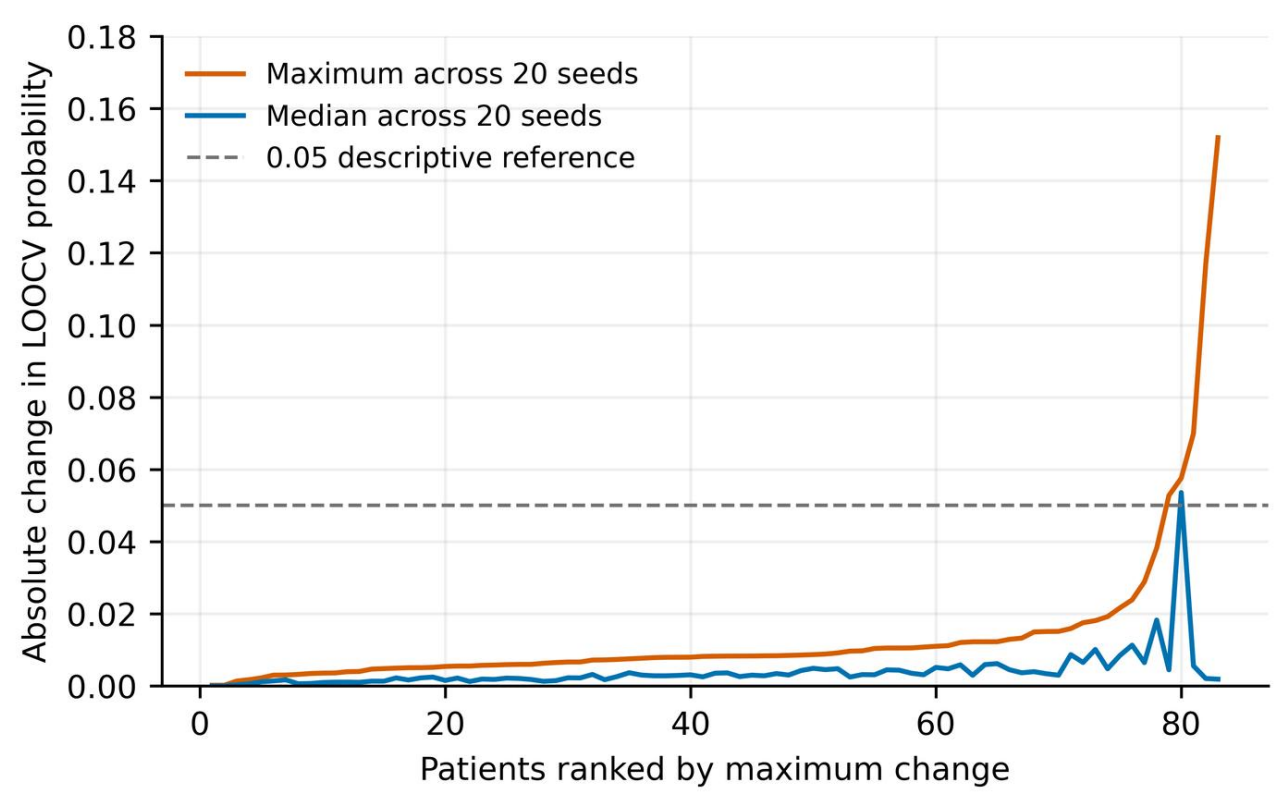

Supplementary Figure S2. K-means initialization stability. (a) Partition and feature stability across 20 fixed alternative K-means random states (0–19), with random state 42 as the locked reference and n_init=10 for every fit. For each of 83 patients, internal panel A shows the minimum adjusted Rand index (ARI), internal panel B shows the minimum habitat-specific Dice coefficients, and internal panels C and D show the largest absolute changes in Hab1 ADC and Hab3 volume proportion, respectively. The 0.90 line is descriptive, not a validated quality threshold. (b) Fixed-feature LOOCV AUC and Brier score for the two-feature model under each alternative seed; dashed lines show the seed-42 reference. Each model used the same 83 patients, including 38 high-Ki-67 events. (c) Patient-level absolute LOOCV probability changes relative to seed 42. Patients are ranked by their maximum change across the 20 alternative seeds; the orange and blue lines show the maximum and median change, respectively. The 0.05 line is a descriptive reference rather than a clinical decision threshold. Seeds are repeated algorithmic runs and were not treated as independent subjects.

Supplementary Figure S3 | Feature-set and scaling sensitivity

a

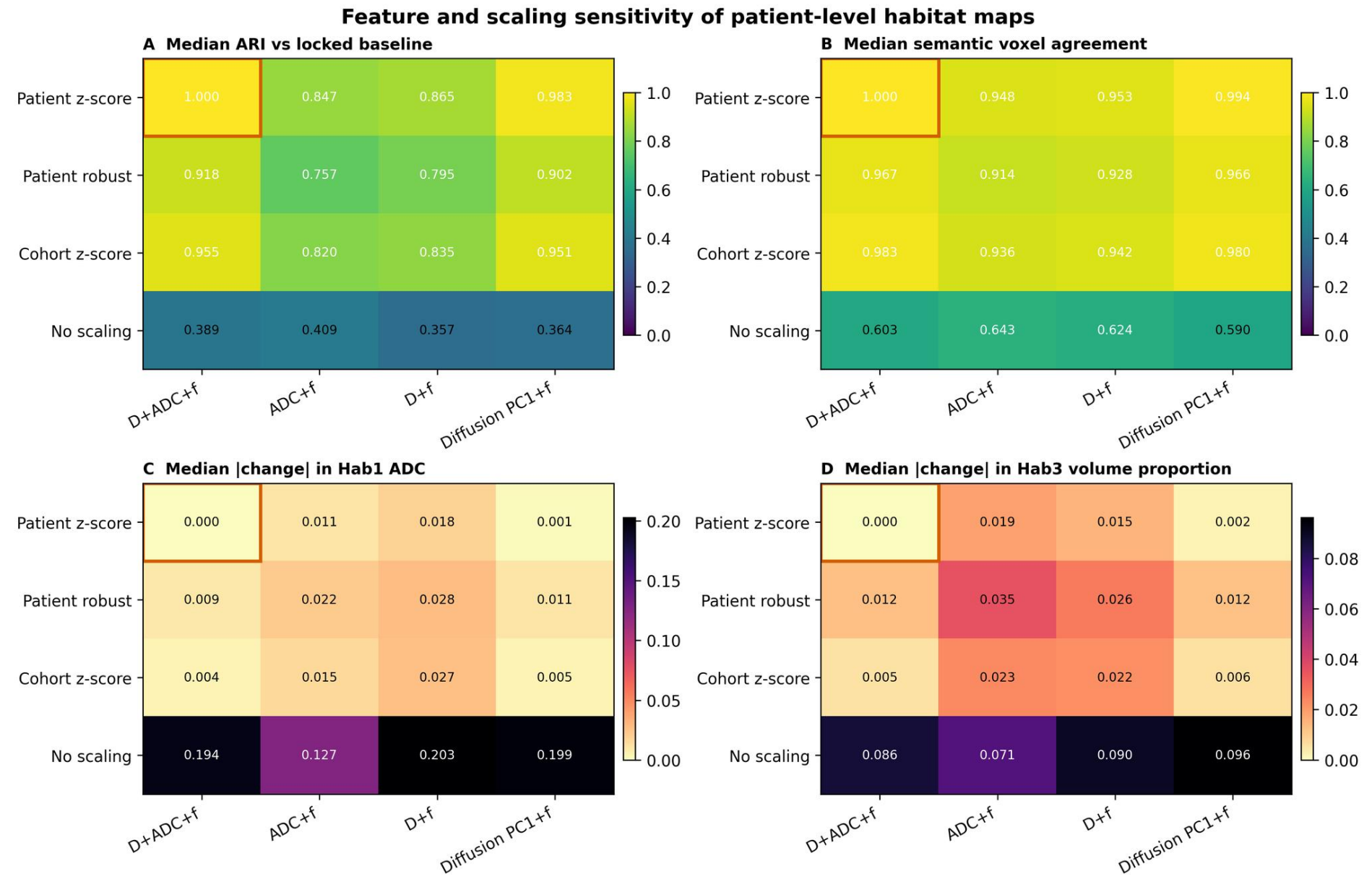

b

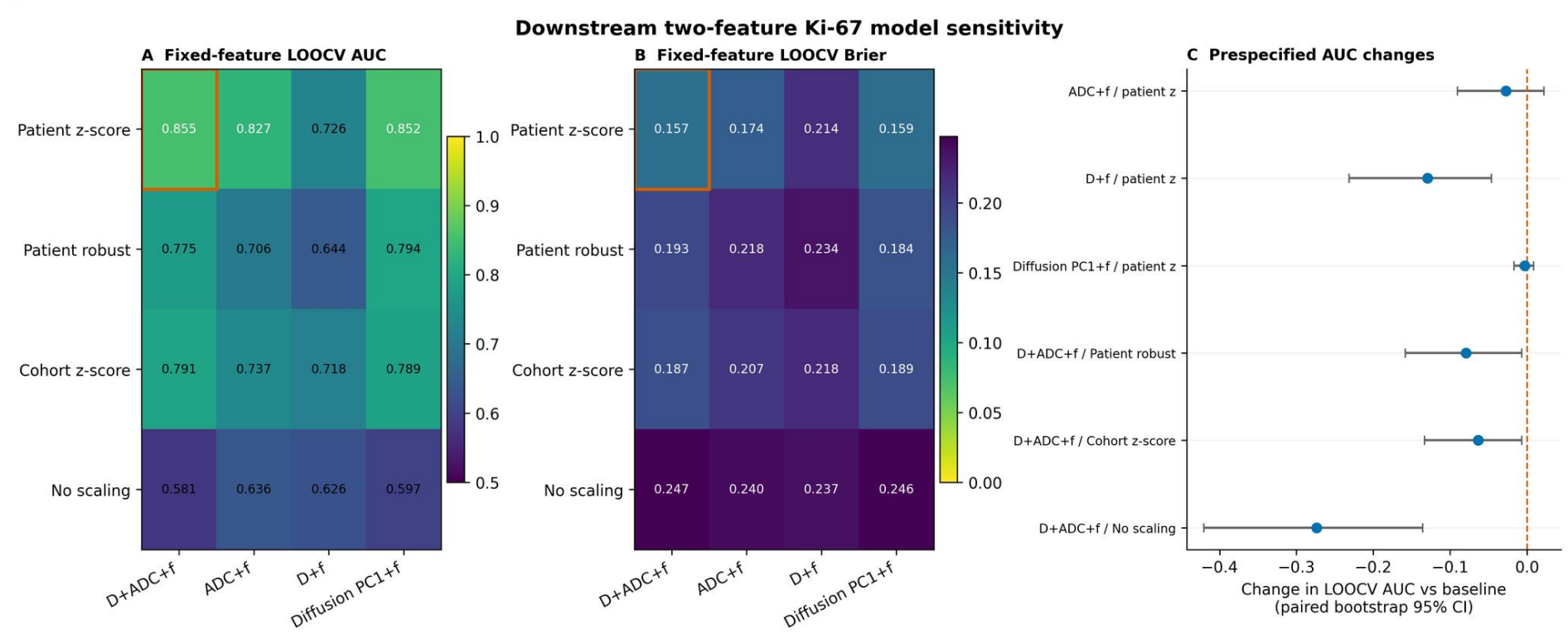

Supplementary Figure S3. Feature-set and scaling sensitivity. (a) Median partition agreement, semantic voxel agreement, and absolute changes in Hab1 ADC and Hab3 volume proportion across four outcome-blind feature sets (D+ADC+f, ADC+f, D+f, and diffusion-PC1+f) and four scaling methods (patient-level z-score, patient-level median/interquartile-range scaling, cohort-level z-score, and no scaling). The orange outline marks the locked D+ADC+f/patient-level-z-score pipeline. (b) Downstream fixed-feature LOOCV AUC, Brier score, and paired AUC changes for the same sensitivity grid. Paired AUC intervals are patient-bootstrap 95% confidence intervals. All summaries use the same 83 patients; cohort-level scaling was not nested inside each held-out fold. The figure is an internal analytical sensitivity analysis and does not imply that the tested representations are interchangeable or universally optimal.

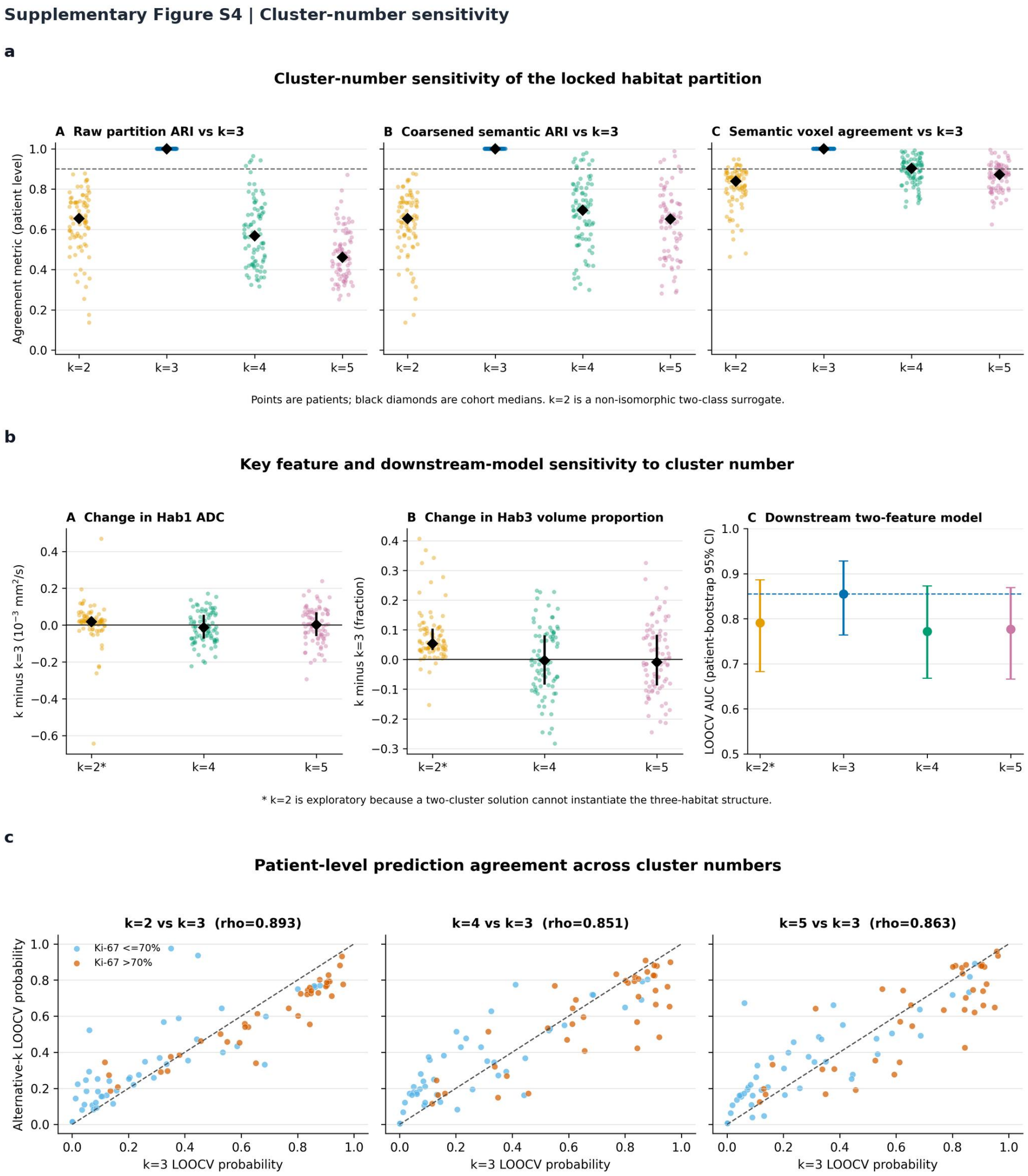

Supplementary Figure S4. Cluster-number sensitivity. (a) Patient-level raw partition ARI, outcome-blind coarsened semantic ARI, and semantic voxel agreement at k=2, 3, 4, and 5 relative to the locked k=3 solution. Points are patients, black diamonds are cohort medians, and the 0.90 line is a descriptive reference. (b) Patient-level changes in Hab1 ADC and Hab3 volume proportion relative to k=3, together with fixed-feature LOOCV AUC and patient-bootstrap 95% confidence intervals. Black diamonds and vertical bars show medians and interquartile ranges for the feature changes. The k=2 result is an exploratory non-isomorphic two-class surrogate because it cannot instantiate the three-habitat structure. (c) Paired patient-level LOOCV probability agreement between each alternative-k analysis and k=3; dashed lines denote identity and the panel titles report Spearman correlation coefficients. For k=4 and k=5, clusters were coarsened to the three locked semantic classes by outcome-blind maximum voxel overlap. These results support k=3 as a fixed working solution in this cohort, not as a universally optimal cluster number.
